# Comparative Sex Chromosome Genomics in Snakes: Differentiation, Evolutionary Strata, and Lack of Global Dosage Compensation

**DOI:** 10.1371/journal.pbio.1001643

**Published:** 2013-08-27

**Authors:** Beatriz Vicoso, J. J. Emerson, Yulia Zektser, Shivani Mahajan, Doris Bachtrog

**Affiliations:** Department of Integrative Biology, University of California Berkeley, Berkeley, California, United States of America; Institute of Science and Technology Austria (IST Austria), Austria

## Abstract

Snakes exhibit genetic sex determination, with female heterogametic sex chromosomes (ZZ males, ZW females). Extensive cytogenetic work has suggested that the level of sex chromosome heteromorphism varies among species, with Boidae having entirely homomorphic sex chromosomes, Viperidae having completely heteromorphic sex chromosomes, and Colubridae showing partial differentiation. Here, we take a genomic approach to compare sex chromosome differentiation in these three snake families. We identify homomorphic sex chromosomes in boas (Boidae), but completely heteromorphic sex chromosomes in both garter snakes (Colubridae) and pygmy rattlesnake (Viperidae). Detection of W-linked gametologs enables us to establish the presence of evolutionary strata on garter and pygmy rattlesnake sex chromosomes where recombination was abolished at different time points. Sequence analysis shows that all strata are shared between pygmy rattlesnake and garter snake, i.e., recombination was abolished between the sex chromosomes before the two lineages diverged. The sex-biased transmission of the Z and its hemizygosity in females can impact patterns of molecular evolution, and we show that rates of evolution for Z-linked genes are increased relative to their pseudoautosomal homologs, both at synonymous and amino acid sites (even after controlling for mutational biases). This demonstrates that mutation rates are male-biased in snakes (male-driven evolution), but also supports faster-Z evolution due to differential selective effects on the Z. Finally, we perform a transcriptome analysis in boa and pygmy rattlesnake to establish baseline levels of sex-biased expression in homomorphic sex chromosomes, and show that heteromorphic ZW chromosomes in rattlesnakes lack chromosome-wide dosage compensation. Our study provides the first full scale overview of the evolution of snake sex chromosomes at the genomic level, thus greatly expanding our knowledge of reptilian and vertebrate sex chromosomes evolution.

## Abstract 2

Analysis of the genomes and transcriptomes of snake species with homomorphic and heteromorphic sex chromosomes reveals the evolutionary dynamics of sex chromosome differentiation.

**Author summary**

Sex chromosomes have evolved from non-sex-determining chromosomes (autosomes) multiple times throughout the tree of life. In snakes, females are the heterogametic sex, in that they have two different sex chromosomes, Z and W, while males have two Z chromosomes. However, while in some snake species (e.g., boids) the Z and W chromosomes look identical (“homomorphic”), in others (e.g., vipers) they are very different in size and structure (“heteromorphic”); yet other species (e.g., colubrids) appear intermediate between these two states. This diversity makes snakes ideally suited for studying the evolutionary dynamics of sex chromosome differentiation. Here we sequence the genomes of three snake species (boa, rattlesnake, garter snake) that display varying levels of sex chromosome heteromorphism and perform a comparative genome analysis. This allows us to establish important principles in the biology of snake sex chromosomes, including the identification of evolutionarily distinct regions along the snake sex chromosomes that reflect the loss of recombination at different time points, higher mutation rates in male snakes, and faster evolution of protein-coding genes on snake Z chromosomes. In contrast to conclusions drawn from previous cytogenetic data, we show that the sex chromosomes of colubrid snakes are completely heteromorphic at the DNA sequence level, and that recombination along the sex chromosomes was already abolished in a common ancestor of colubrids and vipers. Finally, we also show that snakes have not evolved chromosome-wide mechanisms to compensate for reduced gene expression of the sex chromosomes in the heterogametic sex.

## Introduction

Heteromorphic sex chromosomes are derived from ordinary autosomes [1],[2]. The acquisition of a sex-determining gene initiates sex chromosome evolution, and in many lineages, initially identical homomorphic sex chromosomes differentiated into heteromorphic sex chromosomes [1],[2]. For homomorphic sex chromosomes to evolve independently and differentiate, recombination needs to be abolished between the homomorphic proto-sex chromosomes. Sexually antagonistic alleles that accumulate on proto-sex chromosomes are thought to select for a suppression of recombination between homomorphic sex chromosomes [2]–[4]. A lack of recombination, over parts or all of the length of the sex chromosome that is limited to one sex only (the Y in species with male heterogamety, or the W in species with female heterogamety) results in degeneration of the non-recombining region. That is, the non-recombining portion of the Y or W chromosome loses most of the genes that were ancestrally present on the proto-sex chromosome [4]. Differentiation of homomorphic sex chromosomes resulting in heteromorphic sex chromosomes is a by-product of degeneration of non-recombining regions along the Y or W chromosome, and can encompass the entire chromosome, or just parts of it. Many familiar sex chromosome systems, such as those of humans or *Drosophila*, have highly differentiated X and Y chromosomes, exhibiting few signatures of their evolutionary history [4]. Some species groups, however, contain taxa at various stages in this transition from morphologically identical to fully differentiated sex chromosomes. Why some species abolish recombination along their sex chromosomes and acquire heteromorphic XY or ZW chromosomes, while others maintain homomorphic sex chromosomes, is not entirely clear. In principle, this could be due to a lack of sexually antagonistic mutations in some species, or the resolution of conflict imposed by sexually antagonistic mutations by evolving sex-specific or sex-biased expression [5].

All snakes exhibit genetic sex determination, with females being the heterogametic sex, but cytogenetic studies suggest that tremendous variation exists among taxa with regards to the level of W degeneration [6],[7]. It was in fact comparative cytogenetic work in snakes that led Ohno to first propose that vertebrate sex chromosomes are derived from autosomes, and snakes show the entire continuum of sex chromosome differentiation [6],[7]. Boidae and Pythonidae, which diverged from the remaining snakes about 100 MY ago [8], have homomorphic sex chromosomes, that is the Z and W chromosomes appear undifferentiated at the cytological level. In contrast, W chromosomes appear highly degenerated and heterochromatic in snakes belonging to the Elapidae and the Viperidae. Colubrid snakes, which diverged from vipers about 50 MY ago [8], are at an intermediate stage of sex-chromosome evolution and often have moderately differentiated Z and W chromosome karyotypes [6],[7],[9],[10]. Thus, snakes are an excellent model system for studying the evolutionary processes driving sex-chromosome differentiation in vertebrates. In addition, snakes provide an independent example of a female-heterogametic system. Like in birds, female snakes contain the sex-limited chromosome, and while ZW systems mimic XY species in many respects, such as in the degeneration of the non-recombining W or Y chromosome, they appear to differ in other aspects [11],[12]. In particular, while XY species generally have global dosage compensation mechanisms that result in equal expression of X-linked genes in males and females, all ZW species studied to date lack chromosome-wide dosage compensation [13]–[17]. The generality of these patterns, and the evolutionary reasons for this different behavior of XY versus ZW systems, however, are not fully understood [12],[18].

Thus, while snakes promise to reveal important insights into sex chromosome differentiation, the lack of genomic resources has limited progress. Here we sequence and perform a comparative genome analysis of three snake species displaying varying levels of sex chromosome heteromorphism to investigate sex chromosome differentiation in this clade. In boas (*Boa constrictor*, family Boidae), the Z and W are mostly homomorphic and show low levels of differentiation, while pygmy rattlesnakes (*Sistrurus miliarius*, family Viperidae) have highly differentiated sex chromosomes with little to no homology remaining between the Z and W [6],[7],[9],[19]. Garter snakes (genus *Thamnophis*, family Colubridae) contain species with both homomorphic and heteromorphic sex chromosomes [20], and we sequenced the common garter snake (*Thamnophis elegans*), which is reported to have homomorphic sex chromosomes based on cytological evidence [20]. In addition, we obtain transcriptomes from boa and pygmy rattlesnake, to test for dosage compensation in snakes. Comparing the transcriptomes between male and female Boidae allows us to establish baseline levels of sex-biased transcription at homomorphic sex chromosomes, to test for the absence or presence of dosage compensation at heteromorphic sex chromosomes in Viperidae.

## Results

### Genome and Transcriptome Assemblies in Snakes

We sequenced the genome of a single male and single female of the common garter snake (*T. elegans*, family Colubridae) and pygmy rattlesnake (*S. miliarius*, family Viperidae), as well as a single female boa (*B. constrictor*, family Boidae; publically available male boa reads and scaffolds were obtained from the Assemblathon website, see Methods). Pygmy rattlesnake and garter snake scaffolds were assembled using SOAPdenovo (Table S1). We performed RNA-seq on a single male and female individual of both boa and pygmy rattlesnake to study patterns of gene expression (see Methods). A SOAPdenovo assembly yielded 297,551 transcripts for boa and 142,100 for pygmy rattlesnake, of which 43,977 and 37,667, respectively, mapped to protein-coding genes of the lizard *Anolis carolinensis*, the closest reptile relative with a completely sequenced and annotated genome. After removal of redundant transcripts and concatenation of transcripts corresponding to different parts of the same *Anolis* gene, our final sample consisted of 10,793 boa genes and 11,939 pygmy rattlesnake genes.

### Identification of Z-Linked Sequences in Snakes

Karyologically, snakes are highly conserved with a preponderance of species with 2n = 36 (16 macro- and 20 microchromosomes). All snakes show genetic sex determination, with females being ZW. The sex chromosomes of snakes were shown to be homologous in different families [9],[10], and correspond to chromosome 6 of the butterfly lizard *Leiolepis reevesii rubritaeniata* [21]. Karyotypes and synteny have been well conserved during 280 million years of reptile evolution; for example, 19 out of 22 anchored chicken chromosomes are syntenic to a single *Anolis* chromosome over their entire length [22]. Thus, we used chromosomal information from *Anolis* as a proxy to anchor genes along chromosomes in snakes.

**Figure 1:**
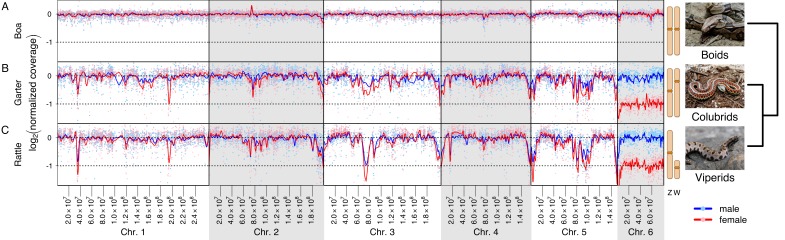
Normalized read coverage depth for female (red) and male (blue) scaffolds ordered along the *Anolis* genome for (A) boa, (B) garter snake, and (C) pygmy rattlesnake. The points show the normalized log_2_ coverage for each scaffold and the lines represent a smoothing spline drawn along the chromosome. Coverage was normalized by dividing the coverage for each scaffold-by-sex combination by the median coverage of all scaffolds in chromosomes 1–5 in that sex, resulting in a median log_2_ coverage score for autosomes of 0. Under this normalization, hemizygous sequences are expected to have a median log_2_ coverage of −1. Scaffolds were mapped to the *Anolis* macrochromosomes on the basis of the location of their gene content. The phylogenetic relationship between the species investigated is shown. Boids split from the other two groups about 100 MY ago, while colubrids and viperids diverged about 50 MY ago [8]. The snake photographs are used under a Creative Commons Attribution 2.5 Generic license (CC BY 2.5). Credit for the photographs are as follows: Nick Turland for the western pygmy rattlesnake (http://www.flickr.com/photos/nturland/1436776818/); Guilherme Jófili for the Boa constrictor (http://www.flickr.com/photos/gjofili/5005623645/); Steve Jurvetson for the coastal garter snake (http://www.flickr.com/photos/jurvetson/825514494/). Additional permission to publish the western pygmy rattlesnake image was granted by Nick Turland.

We used genomic coverage of *de novo* assembled scaffolds in males versus females to identify sex chromosomes of snakes. Specifically, Z-linked regions with a degenerate W homolog should only have half the genomic coverage in females relative to males, while autosomal regions and undifferentiated sex-linked regions (pseudoautosomal regions [PARs]) should have equal genomic coverage in both sexes [14]. Genomic scaffolds from all three species were assigned to the chromosomes of *Anolis*, and male and female genomic reads were mapped to the scaffolds to estimate their male and female coverage. This coverage analysis reveals that snake scaffolds homologous to chromosomes 1–5 of *Anolis* have similar coverage in males and females in all three species (Figure 1). Chromosome 6 of *Anolis* corresponds to the Z chromosome of pygmy rattlesnake and garter snake, as it shows an almost 2-fold reduction in female to male coverage relative to the other chromosomes in this species (Figure 1). No such reduction is observed in boa, confirming that the Z and W are homomorphic even at the sequence level in this species. Thus, the coverage analysis shows that, at the nucleotide level, both pygmy rattlesnake and garter snake have fully differentiated sex chromosomes (Figure 1); we do not identify any segments along the Z chromosome with similar coverage in the two sexes, as would be expected for a PAR. Note that we cannot rule out the presence of very small PARs, or that the PAR is derived from a region not homologous to *Anolis* chromosome 6. On the other hand, the sex chromosomes of boa appear entirely homomorphic, and we detect no regions of differentiation along the Z, indicating that the boa sex chromosome is indeed recombining over almost all of its length (see Discussion).

### Verification of Z-linkage and Conservation of Synteny

Two independent lines of evidence confirm that we correctly assign sex-linkage of our genomic scaffolds. First, SNP patterns in our RNA-seq sample corroborate that homologues of *Anolis* chromosome 6 genes are Z-linked in snakes (the higher coverage of the RNA-seq data for many genes makes SNP inferences more reliable than using our genomic data). If the W is fully degenerated, as in pygmy rattlesnake, SNP patterns are expected to differ between males and females on the Z but should be similar on autosomes. Female individuals carry only one copy of the Z, and should therefore harbor no polymorphism at Z-linked genes, whereas ZZ male individuals can exhibit variation at Z-linked loci. Consistent with this, over 25% of all pygmy rattlesnake genes assigned to chromosomes 1 to 5 have at least one SNP in both the male and female sample. For Z-linked genes (chromosome 6), this proportion is 29% for the male, but only 2% for the female (7 genes out of 247; see Figure S1), confirming that overall we are classifying sex-linked genes correctly. The seven genes with female SNPs are not adjacent to each other on the Z, and thus they are unlikely to be derived from an undetected PAR. Instead, remaining heterozygosity could be due to several factors, including sequencing or mapping errors, mapping of W-derived reads (see next section), undetected paralogs, or movement of these genes to an autosome. In boa, the W and Z are homomorphic; thus, most Z-linked genes have a homologous copy on the W and are therefore expected to show normal levels of polymorphism in the female. This is indeed what we observe, with female SNP counts of genes on the boa Z well within the range of the autosomes (Figure S1).

Second, we mapped 11 known Z-linked cDNA clones of the rat snake *Elaphe quadrivirgata* [9] to the *Anolis* genome, and nine of them mapped to chromosome 6 of *Anolis* (the remaining two mapped to unmapped scaffolds; Figure S2). On the other hand, only one of the remaining non-sex-linked clones of rat snake mapped to chromosome 6 while 92 mapped to other chromosomes or to unmapped regions (Table S2). This strongly suggests that the Z chromosome of snakes is fully homologous to the *Anolis* chromosome 6. The 11 Z-linked markers were found to be collinear in three snake species investigated by FISH mapping (with representatives from each of Boidae, Viperidae, and Colubridae [9]), and we found the same order of these markers along chromosome 6 of *Anolis* (Figure S2). Thus, the overall structure of chromosome 6 of *Anolis* and the Z of snakes appears conserved, suggesting that there have been no large-scale chromosomal rearrangements between snakes and lizards. In addition, we also compared synteny of the genome assemblies of *Anolis* and boa, the snake species with the best-assembled genome, to detect chromosomal rearrangements at a smaller scale. Indeed, we find that micro-synteny is also remarkably conserved between these two species; almost all of the boa scaffolds are co-linear with *Anolis* (Figure S3), and we find evidence for only two small inversions on the Z chromosome of boa. Thus, all genes in our snake data mapping to chromosome 6 of *Anolis* were assigned as Z-linked, while genes mapping to chromosomes 1 to 5 were used as autosomal control genes. In addition to conserved chromosomal homology, we also expect that the relative locations of genes along the Z chromosome are, to a large extent, conserved between species (Figure S2).

### Identification of W-Linked Sequences in Snakes

**Figure 2:**
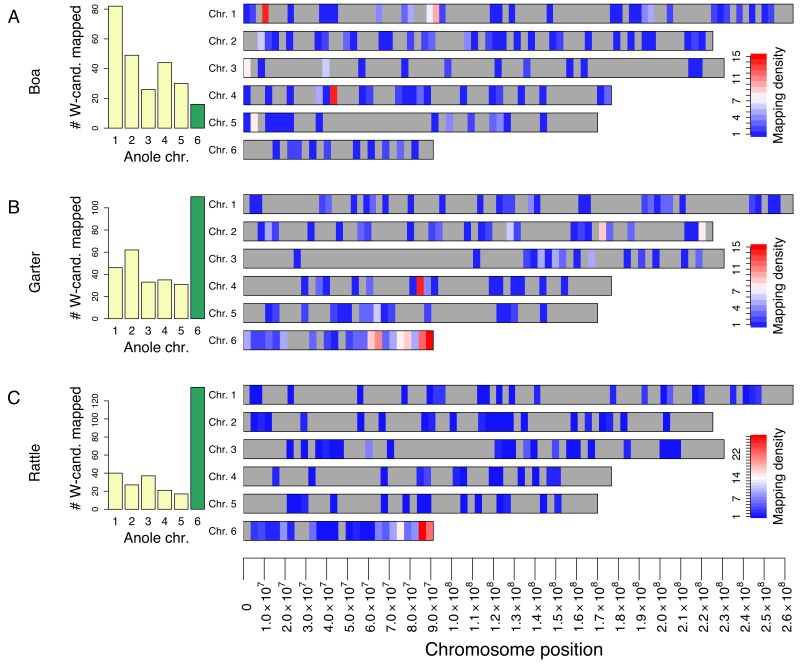
Mapping of the best candidate female-specific W-candidate scaffolds of (A) boa, (B) garter snake, and (C) pygmy rattlesnake to the *Anolis* macrochromosomes. The histograms (left) show the number of candidate W scaffolds mapped to the six major chromosomes of *Anolis*, with the green bar highlighting the *Anolis* homolog to the snake Z chromosome. The W candidates homologous to Z-linked scaffolds (which accounts for 7.6% of the genome) make up 49% and 35% of all female-biased scaffolds in pygmy rattlesnake and garter snake, respectively. This is a 6.4-fold excess in pygmy rattlesnake, and 4.5-fold excess in garter snake over random mapping based on chromosome size. In contrast, in Boa, only 6.5% of scaffolds map to chromosome 6, which does not differ from random mapping on the basis of chromosome size. The right panel shows color-coded mapping density of W-candidates along the *Anolis* macrochromosomes. The density of W-candidates is not uniform across the Z chromosome in both pygmy rattlesnake and garter snake (*p*<0.0001 for mean nearest neighbor distances). The data in this figure are from all female biased candidate W-linked scaffolds and will thus contain both non-coding scaffolds as well as scaffolds containing protein coding genes.

W-linked sequences are present only in females, and we searched for genomic scaffolds with female-specific read coverage to identify putative W-derived scaffolds (see Methods). In order to gain a chromosome-wide perspective on conservation of putatively W-linked sequences, we examined female-specific genomic scaffolds, regardless of whether they contained coding regions (putative W-linked coding sequences are discussed in the section Recombination suppression predates Viperidae–Colubridae divergence below). We selected female specific scaffolds that mapped to scaffolds homologous to Anolis chromosomes 1–6 with more than 150 aligned nucleotides as a candidate set enriched for W-derived sequences. If such putative W-linked sequences are derived from genomic regions of the ancestral autosome that formed the sex chromosomes in snakes, we expect them to be enriched for sequences homologous to inferred Z-linked scaffolds (i.e., scaffolds homologous to *Anolis* chromosome 6). Indeed, we find this to be the case: while the Z chromosome homolog in *Anolis* accounts for only 7.6% of the macrochromosomal genome, we find that 49% of the candidate W-scaffolds with homologs in our assembly map to Z-linked sequences in pygmy rattlesnake and 35% in garter snake (*p*<1×10^−15^; Figures 2 (histograms) and S4). Thus, W-linked candidate sequences in snake species with heteromorphic sex chromosomes are significantly enriched for homologs residing on the Z chromosome (4.5-fold for garter snake, 6.4-fold for pygmy rattlesnake). No such excess is seen for the homomorphic sex chromosomes of boa, and female-biased scaffolds map randomly across the genome, as expected for background noise mapping. Only 6.5% of W-candidate scaffolds of boa map to Anole chromosome 6, which is not statistically different than its proportion of euchromatin in the assembly, or 7.6% (binomial test, *p* = 0.63).

### Evolutionary Strata on Snake Sex Chromosomes

**Figure 3:**
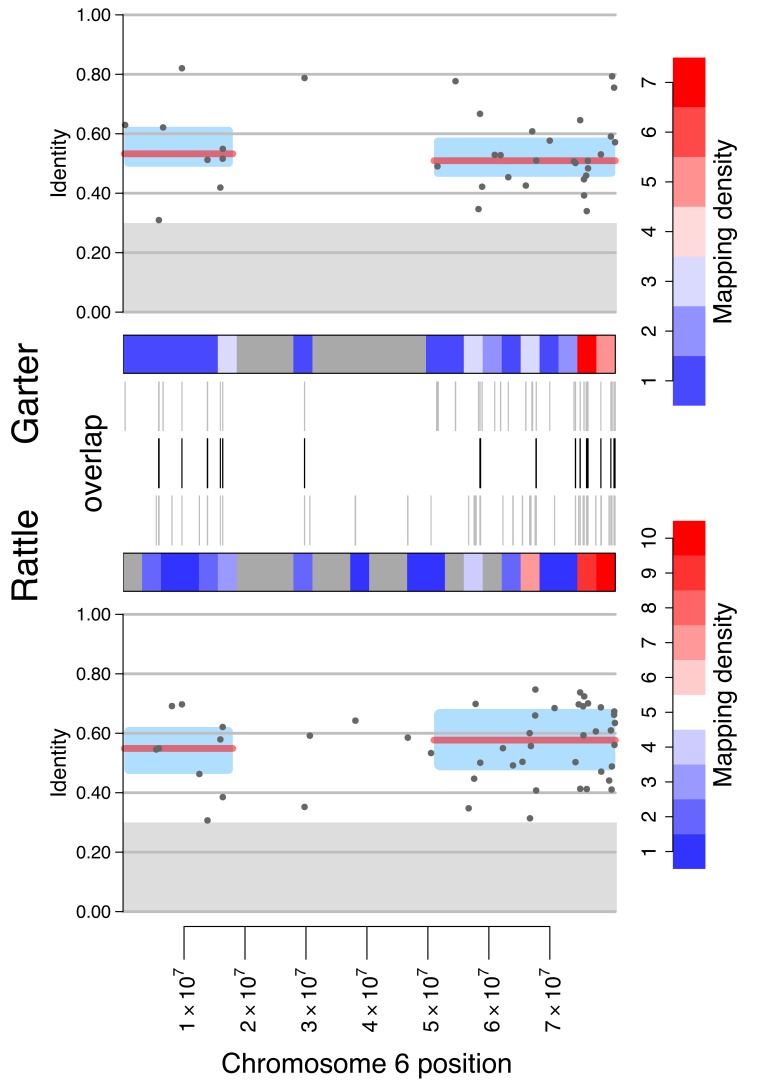
Evolutionary strata and sequence conservation between the pygmy rattlesnake and garter snake W-candidate scaffolds mapped along the Z chromosome. The middle three tracks show the position of candidate W sequences along the Z chromosome in garter snake (top) and pygmy rattlesnake (bottom), and their overlap (center). The top and bottom plots show nucleotide identity between Z-W gametologs (grey dots), and the median (red line) inferred for each of the putative strata for garter snake and pygmy rattlesnake (blue boxes; the y range of the boxes represents the interquantile range of the identity values in each region). The gray shaded region represents identity below 30%, indicating low quality mapping. As in Figure 2, the data in this figure contain both non-coding and coding scaffolds.

Our mapping of these W-candidate genomic scaffolds from each species to their genomic scaffolds anchored along the *Anolis* genome also allowed us to test if W-linked candidate scaffolds are randomly distributed along the Z chromosome, or if they cluster in certain genomic regions. Clustering of W-candidates would suggest that different regions of the Z chromosome have different evolutionary histories, that is, snake Z chromosomes might possess evolutionary strata, as observed in other taxa [23]–[25]. In particular, regions of the Z chromosome with a higher density of W scaffolds and higher similarity between Z and W sequences are indicative of segments along the sex chromosomes that abolished recombination more recently than ones that lack W homologs [23]. Figure 2 shows our mapping of candidate W-linked scaffolds against autosomal and Z-linked scaffolds in each species. We find that the density of W-scaffolds is not uniform across the Z for either pygmy rattlesnake or garter snake (*p*<0.0001; Figure S4). In both pygmy rattlesnake and garter snake, we identify at least two (and possibly three) evolutionary strata across the Z chromosomes that differ in their density of W paralogs, and their degree of nucleotide conservation between the Z and W (Figures 2 and 3). Enrichment of W scaffolds on the Z and identification of strata is robust to the cutoff used to select W-candidates (Figures S5 and S6).

Limited Z-W homology does not allow us to precisely determine the boundaries of strata or their age, but the overall architecture of strata looks similar in pygmy rattlesnake and garter snake. In particular, in both species the oldest stratum encompasses the middle of the chromosome (from roughly 2×10^7^–5×10^7^ bp), with a lower density of homologous sequences between the Z and W, most of which match at levels that are no different than chance (Figure 3). The distal regions on the Z show a much higher density of W-linked candidate regions compared to the center segment (Figure 3; *p*<<0.0001 for both garter snake and pygmy rattlesnake), indicating that the middle of the W chromosome is largely degenerated whereas the distal regions maintain substantial homology. A lower mapping density of the first 20 Mb might be indicative of a slightly older stratum in both pygmy rattlesnake and garter snake than the last 30 Mb (see Figures 2 and 3), but divergence within those regions is comparable (46% versus 42% identity for garter snake and 53% versus 51% for pygmy rattlesnake, neither comparison significantly different). Whether or not the two distal regions represent the same stratum or different strata remains unresolved, and will likely require high quality sequencing of both sexes of multiple species among Colubrids and Viperids. Thus, our data clearly reveal the presence of at least two, and possibly three evolutionary strata on snake sex chromosomes (Figures 3 and S6), and their similar architecture suggests that their formation may have pre-dated the split between Viperidae and Colubridae roughly 50 MY ago.

### Recombination Suppression Predates Viperidae–Colubridae Divergence

Three lines of evidence support that the strata identified in the genomic scaffolds above were indeed formed prior to the divergence between the two species groups. Mapping Z-linked genes to putative W-linked sequence allowed us to retrieve W-gametologs for 55 of these genes in pygmy rattlesnake and 29 in garter snake (see Methods). The average synonymous sequence divergence between the Z-W gametologs exceeds the median synonymous divergence between garter snake and pygmy rattlesnake (0.28 versus 0.20, *p* = 0.076 with a two-tailed Wilcoxon test for pygmy rattlesnake; 0.32 versus 0.20, *p* = 0.004 for garter snake; Figure S7; Table S3, S4, S5), suggesting that recombination between the sex chromosomes ceased before the species split. In addition, if the W chromosomes had diverged completely independently in the two lineages, limited overlap between W-linked sequences (coding and non-coding) between garter snake and pygmy rattlesnake would be expected, but we find an excess of regions along the W to be shared between species (Figures 3 and S8; *p*<0.0001 for all comparisons, via Monte Carlo resampling of observed numbers of scaffolds based on locations of all scaffolds). Similarly, we find more protein-coding genes shared by the W-chromosomes of both species than expected by chance (15 observed versus 1.9 expected, *p*<0.001 with a goodness of fit Chi-square test). Although this fraction is highest for genes in the oldest stratum (Figure S9, due to the presence of a single common gene in this part of the W-chromosomes of both species), it is above 1 for each interval (from 4.6 to 14). This suggests that many W-linked genes had already degenerated in the ancestor of pygmy rattlesnake and garter snake, and that the W had started to reach its equilibrium gene content at the time the two species split, especially in the oldest stratum where recombination was first abolished. Finally, we compared phylogenetic trees for ten regions along the sex chromosomes for which we had homologous sequences from both the pygmy rattlesnake and garter snake Z and W chromosomes. If recombination suppression between the Z and W chromosomes preceded species divergence, we would expect the sequences to cluster by chromosome (i.e., W pygmy rattlesnake with W garter snake, and Z with Z). If pygmy rattlesnake and garter snake abolished recombination at some (or all) of the strata independently, the sequences should cluster by species. We find that for each gene tree constructed, the sequences cluster by chromosome and not species, suggesting that all strata on the sex chromosomes of Viperidae and Colubridae were formed prior to their species divergence (Figure S10).

### Transcribed W-Genes

In addition to identifying 55 W-candidate genes from the pygmy rattlesnake genome assembly, we also assembled the female pygmy rattlesnake RNA-seq reads *de novo*, and searched this assembly for W sequences, based on female-limited expression and female-biased sequence coverage. Briefly, we mapped the pygmy rattlesnake male and female DNA-seq and RNA-seq reads to the transcriptome using Bowtie2 to estimate, for each transcript, the male and female coverage and expression level. While comparisons between female and male liver are unlikely to yield many genes with female-specific functions (as expected on the W chromosome), we find 12 pygmy rattlesnake transcripts with female to male coverage ratio below 0.1 and female-specific expression (nine after excluding transcripts derived from parasites), and W-linkage of six of these candidates was confirmed by female-specific PCR products (Figure S11). The list of confirmed and putative W-linked transcripts is provided in Table S6. Of the six confirmed W-derived transcripts, three consist of repetitive sequences or sequences derived from transposable elements, in agreement with the idea that degeneration of W/Y-chromosomes is often driven by the accumulation of active transposable elements. Although they were expressed at high enough levels to be detected in the female transcriptome, these repetitive/TE sequences had overall lower expression levels (mean fragments per kilobase of transcript per million mapped reads [FPKM]: 6.8) than the three other W-linked transcripts (mean FPKM: 13.4), providing some support for a functional role of the latter. Two of these non-repetitive transcripts have significant similarity to known vertebrate genes (ube2m and 28S ribosomal RNA gene, with tblastx E-values of 3.00e–11 and 1.00e–71), while the third transcript does not map to any known gene or contain any open reading frames (but has a relatively high level of expression at 14.2). Combining the DNA and RNA-based approach, we identify 61 potential genes on the W of pygmy rattlesnake (six from the transcriptome, and 55 from female-specific genomic scaffolds that map to chromosome 6/Z genes) and 29 on the W of garter snake (15 of which overlap between pygmy rattlesnake and garter snake). Thus, the W chromosome in both species contains considerably fewer genes than its former homolog, the Z chromosome (we detect 61 W-linked genes versus 712 Z-linked genes in pygmy rattlesnake, and 29 W-linked genes versus 723 Z-linked genes in garter snake).

### Molecular Evolution of Snake Sex Chromosomes

Sex chromosomes are subject to unique evolutionary forces compared to autosomes [26]. Sex-biased transmission and hemizygosity of the Z in females can impact the efficacy of natural selection on the Z relative to autosomes (faster Z evolution). On one hand, selection can be more efficient at incorporating adaptive recessive mutations on the hemizygous Z-chromosome, resulting in increased rates of adaptive evolution [27],[28]. Alternatively, the Z can also experience more genetic drift due to a smaller effective population size relative to autosomes, resulting in an accumulation of slightly deleterious mutations [29],. In addition, Z-chromosomes are more often transmitted through males than are autosomes, and might be subject to higher mutation rates (male-driven evolution). That is, the many more cell divisions in spermatogenesis than in oogenesis can result in more mutations during DNA replication and thereby skew mutation rates between chromosomes [31]–[33]. Since synonymous sites are generally expected to evolve neutrally in vertebrates, rates of synonymous divergence (K*_s_*) can be used as a proxy to infer mutation rate differences (i.e., male-driven evolution), while rates of amino-acid evolution (K*_a_*) allow inferences regarding the efficacy of natural selection, and this has been used extensively to detect faster-X/Z evolution in a variety of species.

### Higher Rates of Protein and Synonymous Site Evolution on the Z

**Figure 4:**
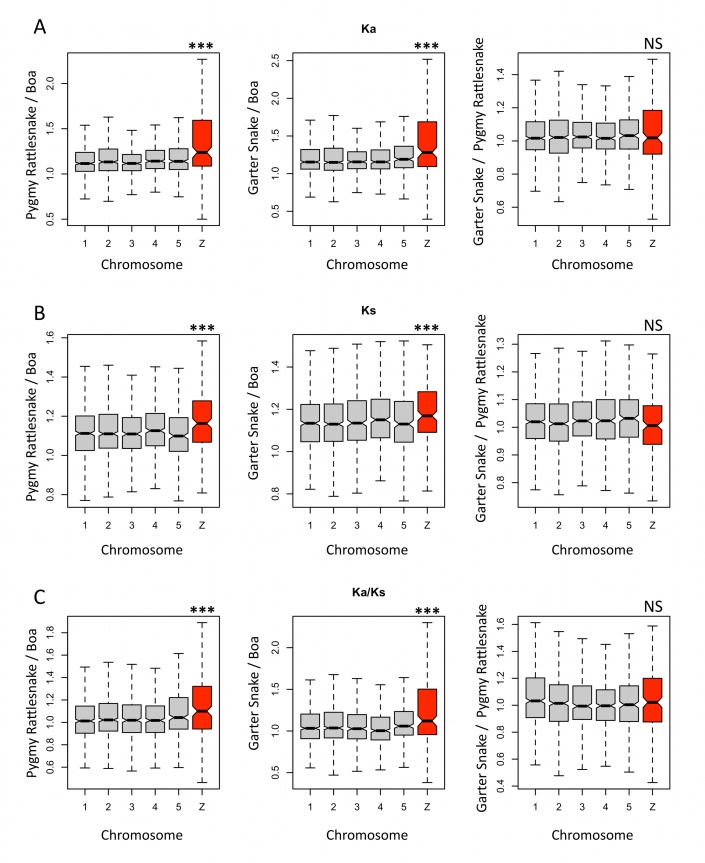
Molecular evolution of snake Z chromosomes and autosomes at synonymous sites (K_s_) and non-synonymous sites (K_a_), and their ratio (K_a_/K_s_). For each gene, we calculated the *Anolis*-boa, *Anolis*-pygmy rattlesnake, and *Anolis*-garter snake rates of synonymous and non-synonymous evolution. To detect branch-specific differences, we obtained for each gene the ratios of these evolutionary rates between the different snake species pairs (pygmy rattlesnake/boa, garter snake/boa, and garter snake/pygmy rattlesnake), and plotted them for each macrochromosome. Please note that in the figure, “Pygmy Rattlesnake/Boa” refers to the ratio: pygmy-*Anolis* divergence/boa-*Anolis* divergence, and so on. Significant differences between the Z-chromosome and the autosomes are marked with asterisks (***, *p*<0.001; NS, non-significant).

The traditional approach to detect faster-X/Z evolution has been to compare rates of evolution of Z/X-linked genes to those of autosomal genes between a pair of species. Using pygmy rattlesnake and garter snake, this approach suggests that the Z is prone to accelerated rates of both synonymous (K*_s_* autosome = 0.17 versus K*_s_* Z = 0.19; see Tables S3 and S7) and non-synonymous (K*_a_* autosome = 0.032 versus K*_a_* Z = 0.037) evolution. However, the results of such pairwise comparisons are highly dependent on the gene content of each chromosome and can yield inconsistent results [34]. In snakes, the same set of genes is present on a differentiated Z (in pygmy rattlesnake and garter snake) and in a pseudoautosomal region (in boa), which is not expected to be subject to faster-Z or male-driven evolution. Under faster Z and male-driven evolution, we therefore expect this set of Z-linked genes to show an accelerated rate of divergence in the lineages with heteromorphic sex chromosomes (pygmy rattlesnake and garter snake) relative to the homomorphic lineage (boa). We calculated pairwise rates of synonymous (K*_s_*) and amino acid (K*_a_*) evolution, as well as their ratio (K*_a_*/K*_s_*), at protein-coding genes between *Anolis* and the different snake species. To test if they were consistent with increased rates of divergence on the Z in pygmy rattlesnake and garter snake compared with boa, we compared the following ratios (K stands for K*_a_*, K*_s_* or K*_a_*/K*_s_* depending on the analysis; see Figure 4) on the Z versus the autosomes: K(garter snake-*Anolis*)/K(boa-*Anolis*), K(pygmy rattlesnake-*Anolis*)/K(boa-*Anolis*), and K(garter snake-*Anolis*)/K(pygmy rattlesnake-*Anolis*). Under faster-Z and male-drive evolution, both K(pygmy rattlesnake-*Anolis*) and K(garter snake-*Anolis*) are expected to be increased on the Z, so that the ratio between the two may not differ significantly between the Z and the autosomes. K(pygmy rattlesnake-*Anolis*)/K(boa-*Anolis*), and K(garter snake-*Anolis*)/K(boa-*Anolis*), on other hand, are expected to be increased on the Z relative to the autosomes because boa is not under faster-Z/male-driven evolution (but garter snake and pygmy rattlesnake presumably are).

Overall, comparisons involving pygmy rattlesnake or garter snake yield slightly higher K*_a_* and K*_s_* values than the boa-*Anolis* comparison; however, this difference is significantly larger for the Z-chromosome (Figure 4) at the K*_a_*, K*_s_*, and K*_a_*/K*_s_* level, in agreement with faster-Z and male-driven evolution (Figure 4). Pygmy-*Anolis* and garter-*Anolis* comparisons, on the other hand, yield similar values for all chromosomes, as expected if both are under faster-Z evolution. The elevated K*_a_* and K*_s_* values on the Z suggest a general increase in mutation rates for Z-linked genes in males (i.e., male-driven evolution). The strength of male-driven evolution can be assessed by comparing the rates of synonymous evolution on the Z versus the autosomes, and α, the male to female ratio of mutation, is equal to (3 * K*_s_*Z−2 * K*_s_*A)/(4 * K*_s_*A−3 * K*_s_*Z) (where K*_s_*A and K*_s_*Z are the rates of synonymous evolution on the autosomes and the Z, respectively, and assuming that synonymous sites are generally neutral). Using data from pygmy rattlesnake and garter snake, we estimate that α is ∼1.8. Importantly, a significantly increased K*_a_*/K*_s_* ratio also supports faster evolution of the protein sequences of Z-linked genes, above the increase due to mutation rate differences (Figure 4). In summary, we find that snake genes on differentiated Z-chromosomes undergo accelerated rates of evolution relative to their homologs on the pseudoautosomal region, both at synonymous sites (consistent with different mutation rates between the sexes) and at protein sequences (likely due to mutational effects, but also due to differential selective effects on female-haploid chromosomes).

### Expression Analysis of Z-Linked Genes in Boa and Rattlesnake

**Figure 5:**
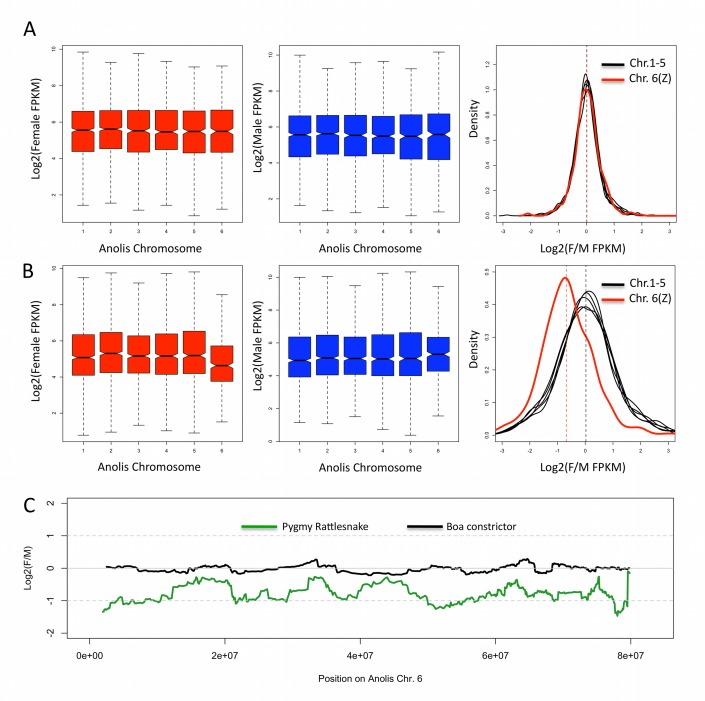
Log_2_ of expression in female, log_2_ of expression in male, and log_2_ of female over male expression, for the different macrochromosomes of boa (A) and pygmy rattlesnake (B). FPKM values were obtained for each gene using Cufflinks. Genes were assigned to different chromosomes according to their location in the *Anolis* genome. (C) log_2_ of female over male expression along chromosome 6 of *Anolis* (Z of snakes), using a sliding window size of 30 genes.

As W-chromosomes lack recombination and degenerate, Z chromosomes may evolve dosage compensation [35]. However, while taxa with male heterogamety generally equalize expression of X-linked genes between the sexes, all species investigated to date in which females are the heterogametic sex appear to lack chromosome-wide dosage compensation [12],[35]. To test for dosage compensation in pygmy rattlesnakes, we assayed gene expression in males and females for Z-linked and autosomal genes. One challenge of studying dosage compensation is that the expectations regarding levels of expression on the Z/X versus the autosomes in females and males are often unclear (see Discussion). Expression in boa provides a clear expectation for snakes, as, in the absence of W degeneration, the Z must have largely maintained the expression levels and biases of the ancestral autosome 6. Figure 5 shows that there is no reduction of expression in either male or female boa for this chromosome (*p* = 0.54, Wilcoxon test), and that the female to male ratio of expression is about 1 for all chromosomes. This provides clear base-line levels of absolute and sex-biased expression when testing for dosage compensation in snake species with heteromorphic sex chromosomes, as any expression bias observed on the Z must have arisen during or after the degeneration of the W.

In pygmy rattlesnake, chromosomes 1–5 have a female to male expression ratio of approximately 1, resembling patterns of gene expression in boas. The Z chromosome (chromosome 6), however, shows a clear reduction in levels of gene expression in females (*p*-value = 5.32e–11, Wilcoxon test), resulting in highly male-biased expression of this chromosome in pygmy rattlesnakes (the median of log_2_(F/M) is equal to −0.71 for the Z, corresponding to a 1.6-fold reduction, versus 0.05 for the autosomes, *p*-value<2.2e–16). While all genes with FPKM >0 were included in this analysis, the patterns are robust to changes in expression cutoffs (Figure S12), and a similar reduction is observed for genes with FPKM >10 in both males and females. This expression analysis thus provides clear evidence of a lack of global, chromosome-wide dosage compensation in ZW snakes with heteromorphic sex chromosomes.

The female Z does not show a full 2-fold reduction of expression, which is likely to be at least partly associated with a general buffering mechanism for gene expression (i.e., a single gene dose does not result in halving of gene transcript), as observed in aneuploid *Drosophila* and human autosomal genes and on the uncompensated regions of the single female chicken Z [12]. In addition, snakes may show local dosage compensation, as found in birds [36], with some Z-linked genes being dosage-compensated. One possibility is that dosage compensation is restricted to segments of the chromosome, as has been suggested for the chicken Z chromosome [37]. A sliding-window analysis of sex-biased expression in pygmy rattlesnakes, with genes mapped according to their position on the *Anolis* chromosome 6, reveals some localized variation in female to male expression ratio along the chromosome, but no segment that is fully compensated (Figure 5). Whether this reflects the biology of the chromosome, or whether compensated chromosomal fragments in pygmy rattlesnake are simply masked by a large number of rearrangements since the snake-lizard split, will require the analysis of a fully assembled genomic sequence. While this is not yet possible for pygmy rattlesnake, a genomic assembly for boa is available (http://assemblathon.org/pages/download-data). Our analysis of synteny of genes on the largest boa scaffolds shows that there is a limited number of rearrangements between snakes and lizards, suggesting that this is unlikely to account for the patterns of female to male expression in pygmy rattlesnake (Figure S13). A more likely possibility is therefore that only some dosage-sensitive genes acquire dosage compensation, and that they do so on a gene-by-gene basis, similar to what is observed in birds and silkworm [12]. Consistent with this, the expression ratio histogram for Z-linked genes in pygmy rattlesnakes shows a shoulder of genes with a log_2_(F∶M) expression ratio of 0 (Figure 5B), in agreement with about 18% of all genes present on the Z being locally compensated. These dosage compensated genes show no clear clustering along the Z (Figure S14), as expected if specific genes become compensated individually on the Z.

## Discussion

### Sex Chromosome Differentiation in Snakes

We compare the genomes of three snake species to study sex chromosomes at different stages of differentiation. Our analysis reveals that boas have entirely homomorphic sex chromosomes with no detectable differentiated segment present on the Z. Several reasons could have prevented us from detecting an existing sex-determining region on the Z/W: first, since coverage data is inherently noisy, a single, small, female-specific region (or a small region of reduced female coverage, if part of the W is degenerated) may not be distinguishable from a false positive. That the Boidae sex-determining region is small is evidenced by the viability of WW boa females produced by facultative parthenogenesis (in snakes with differentiated sex chromosomes, WW females are inviable and only ZZ males or ZW females are thought to result from parthenogenesis) [38],[39]. Another possibility is that the W acquired a sex-determining region from another chromosome, or that part of another chromosome of *Anolis* is fused to the Z/W of snakes. Finally, many genomic scaffolds of *Anolis* remain unmapped, and one of them could correspond to the part of chromosome 6 that has evolved into the sex-determining region of snakes. In contrast to boa, the sex chromosomes of both pygmy rattlesnake and garter snake are completely heteromorphic, and we detect no pseudoautosomal regions in either species (with the caveat that, once again, the PAR could be derived from a region not homologous to chromosome 6 of *Anolis*). Thus, the species investigated appear to represent extreme ends in the process of sex chromosome differentiation, despite cytological data that show little differentiation between the Z and W chromosome of garter snakes [20]. This suggests that one needs to be cautious in the interpretation of cytological data, and many more species that appear to lack sex chromosomes at the cytological level may in fact have differentiated sex chromosomes.

### Old Strata on Snake Sex Chromosomes

Further, we identify dozens of W-candidate sequences in snakes whose closest paralogs in the genome are located on the Z, suggesting that W-sequences, to a large extent, consist of degenerated sequences present ancestrally on the autosomal precursor of the sex chromosomes, and not of recent additions to the W chromosome. The spatial distribution of W-scaffolds along the Z and identities of conserved Z-W sequences indicate the presence of evolutionary strata along the snake sex chromosomes. More recently formed strata show a higher density of W-linked sequences and more similarity with their Z-linked gametologs, compared to older strata. We identify at least two (and possibly three) strata in both pygmy rattlesnakes and garter snakes, and their overall architecture and age distributions are similar in both species. Sequence divergence at Z-W gametologs exceeds that of Z-linked genes between pygmy rattlesnake and garter snake, and comparison of the gene content of W-linked sequences in pygmy rattlesnake and garter snake shows an excess of shared genes between the species. Phylogenetic analysis reveals that W-linked sequences between the two species are more similar to each other than they are to their Z-linked gametologs within a species, indicating that the W-linked regions stopped recombining with their Z gametologs before the species split between Viperidae and Colubridae. All of these data strongly suggest that recombination between the sex chromosomes of pygmy rattlesnake and garter snake was abolished before these two lineages diverged, and all strata were established in an ancestor of Viperidae and Colubridae. Thus, our data are inconsistent with the view that sex chromosomes of snakes have evolved restricted recombination independently in different lineages. Instead, sex chromosomes of both Viperidae and Colubridae have been non-recombining for over 50 MY before their split, and their W-chromosomes are expected to be globally degenerate at the sequence level in both lineages.

### Cytogenetics Versus Degeneration at the Molecular Level

The inferred variation in heteromorphism of sex chromosomes in different snake families from cytological analysis may be driven by general differences in their chromosomal morphology, such as the differential accumulation of repetitive sequences and heterochromatin on the W of different species (which can lead to dramatic differences in size and appearance, as often observed between Y chromosomes of closely related species [40]), rather than reflecting their level of divergence at the DNA sequence level. Similarly, the perceived cytological similarity between the W and Z of some species may stem from small pockets of local identity on otherwise degenerated Ws, as in the case of garter snake. A recent study localizing Z-linked clones using FISH mapping to the chromosomes of the Burmese python (Pythonidae), the Japanese rat snake (Colubridae), and the habu (Viperidae) found that while all 11 Z-linked clones mapped to both the Z and W chromosomes of the Burmese python, only three (RAB5A, CTNNB1, WAC) mapped to the W of the rat snake, and none to the W of the habu [9], supporting the cytological view that Colubridae are at a transitional stage of sex chromosome differentiation and Viperidae having a fully degenerate W chromosome. We searched our W-candidates in pygmy rattlesnake and garter snake for the three genes detected on the rat snake W, and did not detect a W-homolog of RAB5A in either species, found a W-homolog for WAC in both pygmy rattlesnake and garter snake, and identified a W-homolog for CTNNB1 only in pygmy rattlesnake. Thus, while cytological data failed to identify these W-candidate genes in the Viperidae investigated [9], our genome analysis clearly provides evidence for their presence on the W of pygmy rattlesnake, suggesting that the previous observations reflected species-specific gene loss on the W (or difficulties of localizing hybridisation signal on a more heterochromatic chromosome), rather than a general difference in the gene content of the W chromosome of Colubridae and Viperidae.

### Faster Evolution of Snake Z Chromosomes

We find evidence for both male-driven evolution, and faster Z in snakes, similar to patterns observed in birds [29],[32]. Faster Z evolution can be an adaptive or non-adaptive process. On one hand, faster Z evolution may result from increased rates of fixation of recessive beneficial mutations, as Z-linked recessive alleles are directly exposed to selection in females [27]. On the other hand, Z chromosomes might be subject to increased rates of genetic drift [41], because in female-heterogametic taxa the effective population size of the Z can be greatly reduced relative to that of the autosomes, if sexual selection is stronger in males than females [42]. This can lead to an increase in the accumulation of weakly deleterious amino-acid mutations on the Z chromosome [30],[42],[43]. We can use SNP densities in our RNA-seq data to compare effective population sizes between chromosomes. We find that SNP densities are similar for Z-linked and autosomal genes in boas (Z/A ratio: 1.01 in males), but significantly lower in male pygmy rattlesnakes (Z/A ratio: 0.64), reduced below the neutral expectation of 0.75. This is consistent with the polygynous mating system reported in many snakes that can greatly decrease the effective population size of the Z, and suggests that increased drift is likely a main contributor to faster Z in this clade. The difference in rates of molecular evolution for Z-linked genes relative to the autosomes is thus more likely due to neutral and non-adaptive processes in snakes, similar to what has been observed in birds [41].

### Lack of Global Dosage Compensation in Snakes

Our transcriptome analysis provides clear evidence that snakes with heteromorphic sex chromosomes lack global dosage compensation. This result is robust to using different cut-off levels of gene expression, and therefore not an artifact of incorporating lowly expressed genes or transcriptional noise in our analysis [44]. The comparison with boa, which has homomorphic sex chromosomes, allows us to rule out ancestrally lower expression of the Z in females, or an ancestral excess of male-biased genes on the Z. The lack of such a comparison has previously limited our understanding of the evolution of dosage compensation in other clades (but see [45],[46]). For instance, the X-chromosome of the flour beetle *Tribolium castaneum* is over-expressed in females relative to both the autosomes and the male X [47]. This has been interpreted as a primitive form of dosage compensation, achieved by over-expressing the X in both sexes, but could alternatively be understood as a lack of dosage compensation of an ancestrally over-expressed X chromosome. Likewise, Z-linked expression is reduced relative to the autosomes in both sexes in the Lepidoptera *Bombyx mori* [48] and, since the Z is not male-biased in expression, this has been interpreted as evidence for dosage compensation [36]. However, assuming similar ancestral expression of the Z and autosomes, this implies that dosage compensation evolved in this clade by a reduction of Z-linked expression in males, a paradoxical explanation if dosage compensation arises primarily to re-establish Z:autosome gene balance in females. Alternatively, this reduced expression may reflect an ancestral bias that predates sex linkage. Potential sex-biases in expression levels on the Z/X have generally been understood as a lack of dosage compensation, but could also reflect ancestral sex-biased expression of the Z/X, a possibility that cannot be excluded without assaying ancestral gene expression [45],[46]. The contrast between homomorphic and heteromorphic sex chromosomes in snakes eliminates all these confounding possibilities.

The similar expression levels observed for the homomorphic sex chromosome in male and female boas imply that Z-linked gene dose was indeed reduced in female pygmy rattlesnakes following the differentiation of their sex chromosomes, supporting the view that the male-biased expression often observed on Z-chromosomes likely stems from a lack of chromosome-wide dosage compensation. In contrast, most (but not all) species with old heteromorphic XY-systems have evolved mechanisms to counter-balance gene dose differences at sex-linked genes between males and females. XY species that show clear evidence of independent evolution of dosage compensation include marsupials [45], *Drosophila* [44], *Anopheles* [49], and *Caenorhabditis elegans* [44]. Placental mammals inactivate one of their X-chromosomes in females and thus show equal expression of X-linked genes in both sexes [44],[45],[50]. However, most genes on the X are not hyper-expressed relative to ancestral expression levels [45],[46],[51], and instead, some autosomal genes in expression networks with X-linked genes may have been down-regulated in placental mammals to restore gene balance between X-linked genes and autosomes. XY monotremes, in contrast, lack chromosome-wide dosage compensation [45]. *Silene latifolia*, a plant with young XY chromosomes that are only partially degenerated, shows partial dosage compensation [52]. Thus, many—but not all—XY species have evolved dosage compensation. Yet, all species investigated to date in which females are the heterogametic sex appear to lack global dosage compensation [12],[35]. This is true in three broad taxonomic groups with independently evolved ZW chromosomes: birds [13],[16],[53],[54], butterflies [17],[55],[56] (but see [48]), and Schistostomes (a trematode [14]). Snakes with heteromorphic sex chromosomes provide yet another example of a ZW species that lacks a chromosome-wide dosage compensation mechanism.

### Dosage Compensation and Differences in XY Versus ZW Systems

The reason for this consistent difference in male versus female heterogametic systems in whether dosage compensation evolved or not is unclear but several hypotheses could explain the general lack of dosage compensation in female heterogametic species. One possibility is that females are more robust to differences in gene dose than males, and simply do not require compensatory mechanisms [12]. However, it is unclear why this should be the case in such diverse species groups, such as snakes, birds, butterflies, and schistostomes. Alternatively, if the male-biased transmission of the Z has led to a male-specific gene content on this chromosome, dosage compensation in ZW females may in fact be deleterious. The chicken Z chromosome consists of multiple strata that have been sex-linked for different amounts of time [24],[57], and genes that are located in the older evolutionary strata of the Z show more male-biased expression than genes in younger strata [58]. This demonstrates that the gene content of the Z chromosome is becoming more male-biased over time. Why the X, which is similarly expected to become enriched for female-biased genes, should acquire global dosage compensation in XY taxa is, again, not entirely clear, although theory predicts that female-heterogametic systems may be more prone to accumulating sexually antagonistic mutations on the Z than the X in XY species (reviewed in [59]).

While female-heterogametic species lack global dosage compensation, many Z-linked genes are expressed at similar levels in females and males, suggesting that dosage-sensitive genes are up-regulated in females in a gene-specific manner [36]. The propensity to evolve chromosome-wide versus gene-specific mechanisms of dosage compensation could also depend on the number of genes that need compensation simultaneously during the process of Y or W degeneration. Specifically, if Y chromosomes degenerate much quicker than W chromosomes, then several genes simultaneously on the X might require dosage compensation and chromosome-wide mechanisms might evolve [18],[35]. In contrast, if W chromosome degeneration proceeds at a much lower pace, there might only be a single dose-sensitive gene at a time on the Z that requires compensation, and gene-specific dosage compensation might evolve. Higher mutation rates in males, and more opportunity for sexually antagonistic or sex-specific selection in males might result in faster gene decay for Y chromosomes relative to the W, but this has not been tested yet. Finally, it has been suggested that chromosomes that carry genes that are dosage-insensitive may be predisposed to becoming sex chromosomes [60]. Interestingly, in our boa expression analysis, chromosome 6 has the largest standard deviation of FPKM in both male and female (*p*-value<2.2e–16 for F-tests between chromosome 6 and any of the other macrochromosomes; see Dataset S1). This may reflect an ancestral deficit of tightly regulated genes on this chromosome, although it is possible that the increased variance in gene expression is instead a consequence of its sex-linkage. Some support for the idea that the initial set of genes on the proto-sex chromosomes determines the emergence of compensation mechanisms comes from comparison of platypus and chicken; both have homologous sex chromosomes and neither has dosage compensation [45]. In fact, platypus is to date the only species with a non-dosage-compensated XY system, further strengthening this hypothesis.

Another possibility for this apparent difference in XY versus ZW systems is that patterns of sex chromosome dosage compensation might mainly depend on potentially fortuitous events that arose soon after sex chromosome differentiation (e.g., recruitment of MSL complex in Drosophila), which determine the evolution of subsequent mechanisms to equalize expression between the sexes [45]. To distinguish between these hypotheses, one needs to characterize dozens of independently evolved male- and female-heterogametic species with a variety of life-history and population parameters. The application of next-generation sequencing techniques in non-model species to identify sex-linked genes and sex-specific gene expression, as done here, provides a powerful framework to make this feasible.

## Materials and Methods

### Samples

We obtained genome sequences from *B. constrictor* (Boidae), Florida pygmy rattlesnake *S. miliarius barbouri* (Viperidae), and garter snake *T. elegans* (Colubridae) and transcriptomes from *B. constrictor* (blood) and *S. miliarius barbouri* (liver). *B. constrictor* female blood was provided by Monica Albe at UC Berkeley and used for DNA and RNA isolation. DNA and RNA from *B. constrictor* male blood was provided by Mark Stenglein at UC San Francisco. *S. miliarius* livers from a male and a female were collected by Bob Walton and immediately placed in RNAlater, shipped on dry ice and stored at −80°C until RNA and DNA isolation. *T. elegans* male and female livers were provided by the Museum of Vertebrate Zoology at UC Berkeley and used for DNA isolation. See Table S8 for an overview of the number of RNA-seq and DNA-seq reads generated. All DNA/RNA-seq reads are deposited at http://www.ncbi.nlm.nih.gov/sra under the bioproject accession number SRP026493 (genome data) and SRP026494 (transcriptome data).

### Genome Data and Assembly

Illumina paired-end reads from a male *B. constrictor* were downloaded from the Assemblathon project website (http://assemblathon.org/). For all other samples (male and female pygmy rattlesnake, male and female garter snake, female boa), DNA was extracted from each sample with a Qiagen DNeasy Blood and Tissue kit, following the manufacturer's protocol. Paired-end library preparation and sequencing were performed at the Beijing Genomic Institute. All reads were trimmed before further analysis. The garter snake and pygmy rattlesnake genome was assembled from reads derived from separate male and female libraries using SOAPdenovo with a K-mer size of 31. The *B. constrictor* genome assembly 6C of the Assemblathon project was downloaded from http://assemblathon.org/.

### RNA-seq and Transcriptome Assembly

We performed RNA-seq in male and a female liver of *S*. *miliarius barbouri*, and in male and female blood of *B. constrictor*. RNA was extracted with the Qiagen RNeasy kit according to the manufacturer's protocol. Library preparation and sequencing were performed at the Beijing Genomics Institute (en.genomics.cn).

*A. carolinensis* CDS were downloaded from Ensembl release 67 (ftp://ftp.ensembl.org/) and, for each gene, only the longest CDS was kept. For each snake species, male and female reads were pooled, trimmed and assembled using SOAPdenovo [61] with a K-mer value of 31. Gapcloser was run to further improve the assembly. The resulting transcripts were mapped against *A. carolinensis* CDS sequences, using Blat [62] with a translated query and database. For each transcript, only the match with the highest score was kept. When transcripts overlapped on *A. carolinensis* genes by more than 20 bps, only the transcript with the best alignment score was kept. When the overlap was shorter than 20 bps, or when transcripts mapped to different parts of the gene, their sequences were concatenated. The location of these genes on Release 2 of the *Anolis* genome (http://www.broadinstitute.org/ftp/pub/assemblies/reptiles/lizard/AnoCar2.0/) was used to map the snake genes, and genes mapping to chromosome 6 of *Anolis* were classified as Z-linked.

### Chromosome Coordinate Assignment

*Anolis* genes were mapped to genomic scaffolds for both pygmy rattlesnake and garter snake using translated blat searches between *Anolis* CDS sequences (EnsEMBL) and *de novo* snake contigs. In cases where more than one *Anolis* gene mapped to a snake scaffold, the consensus *Anolis* chromosome was used. Gene coordinates and strandedness from the consensus chromosome were used to position and orient the snake scaffolds. Members of the group of genes from the consensus chromosome were thrown out if they were more distant than 1 Mb from the nearest other neighbor in the group. Strandedness of the contig relative to the *Anolis* chromosome was determined by the consensus of colinearity of genes in the cluster. When only one gene mapped to a contig, its position and orientation was used to position and orient the snake scaffolds. The procedure for ordering and orienting boa scaffolds was the same as above. However, because the boa genome has substantially fewer sequencing gaps than the other snake genomes, we were able to construct boa pseudochromosomes directly by concatenating the genomic scaffolds rather than merely translating coordinates from *Anolis* as we did for garter snake and pygmy rattlesnake.

### Coverage Analysis across Chromosomes

For all snake species, male and female reads were aligned separately to their respective genomes using BWA [63]. Read coverage per scaffold was calculated with bamtools [64]. bamtools was used to count the number of read pairs mapping to each scaffold such that: (1) if both reads from a pair mapped to a scaffold, that read pair was counted only once; (2) if only a single read from a pair mapped, it was also counted once, regardless of whether it was the first or second member of the pair. Thus, each sequenced molecule was counted only once.

### SNP Analysis

Female and male RNA-seq reads were mapped back to the assembled transcriptome using Bowtie [65] with default parameters. Profile files were obtained from the resulting Bowtie map files using Bow2Pro (http://guanine.evolbio.mpg.de/), and only sites with coverage over 10 were kept for further analysis. SNPs were called when sites had two bases with frequency over 0.3 times the site coverage (genes with no sites with sufficient coverage were excluded).

### Mapping of Z-Linked and Autosomal Ratsnake Markers

The sequence of each *E. quadrivirgata* marker used in [9] was downloaded from NCBI (http://www.ncbi.nlm.nih.gov/) and mapped to the Release 2 of the *Anolis* genome using blat with a translated query and database. For each marker, only the location with the best mapping score was kept.

### Identifying W Candidate Scaffolds from Genomic Data

In order to search for W-linked sequences, three approaches were used. First, we identified W-linked scaffolds, both coding and noncoding, in all three genome assemblies to assess the global level of differentiation and homology between the Z and the W. Second, we searched for W-linked genes in the genomic data in pygmy rattlesnake and garter snake to investigate gene conservation between the Z and the W, and between the species. Finally we identified putatively expressed W-genes from the transcriptome of pygmy rattlesnake.

For the global identification of W-derived scaffolds, all scaffolds from the *de novo* genomic assemblies showing evidence for high female coverage were identified as W-candidates. As it happens, none of these female biased W-candidates were successfully mapped using *Anolis* genes, likely because of the poorer assembly of W-linked sequences due to their reduced coverage and potentially increased repetitive content, and overall poor conservation of W sequences. As a result, we sought to map W-candidates by searching for their Z-linked homologs, which the W-candidates would have shared a common ancestor with far more recently than with the *Anolis* homologs. Therefore, we homology-searched our W-candidates using lastz and UCSC's axt/chain/net tools [66],[67] against all *de novo* scaffolds that were already successfully mapped to *Anolis* chromosomes as described above (see “Chromosome Coordinate Assignment”). High female coverage was defined with reference to the range of female coverage observed in autosomes (scaffolds homologous to *Anolis* chromosome 1–5) and was determined by selecting scaffolds for which the female proportion of coverage was statistically higher than Q3 + 1.5× IQR on autosomes. We did not initially require female specific read coverage in order to allow detection of incompletely degenerated W-candidate scaffolds. Note that while the homologs of the W-candidates scaffolds were mapped along the Anolis genome based on gene content (see “Chromosome Coordinate Assignment” above), they also contain noncoding sequence. These data are the basis for Figures 2 and 3.

### Identifying W Candidate Genes from Genomic Data

For the genomic search for W-genes (performed in both garter snake and pygmy rattlesnake), male and female genomic reads were mapped to the *de novo* genome assemblies using Bowtie2 with default parameters, and female and male read counts were estimated from the resulting alignments (after removal of alignments with mismatches). Scaffolds with a female read count above 20 and a male read count below 2 were considered as putatively W-linked and kept for further analysis. *Anolis* coding sequences were mapped against these putative W-scaffolds using blat with a translated query and database. As expected, the majority of genes mapping to the putative W-scaffolds are located on *Anolis* chromosome 6/snake Z chromosome (55 Z-linked versus 19 from chromosomes 1–5 in pygmy rattlesnake, 29 versus 23 in garter snake; 42 and 34 were located in unmapped regions in pygmy rattlesnake and garter snake, respectively), and these Z-homologs were classified as putative W-linked genes. The resulting putative W-linked genes were further mapped to their Z-homolog (see “Estimation of K_a_, K_s_ and K_a_/K_s_” for the obtention of snake CDS). For ZW gene pairs with a blat mapping score higher than 100, the coding sequence was extracted manually by aligning both the Z- and W-linked copies of the gene to the corresponding *Anolis* gene with tblastn, and keeping only parts of the sequence for which the protein alignment was well conserved. KaKs_calculator was then run on these ZW gene pairs to estimate their synonymous and non-synonymous rates of evolution (Table S4). For estimating K_s_ between Z/W paralogs using other models of sequence evolution, see Table S7.

### Identifying W Candidate Scaffolds from the Transcriptome

For the identification of putatively expressed W-genes from the transcriptome (performed only in pygmy rattlesnake), female and male RNA-seq and DNA-seq reads were mapped to the *de novo* transcriptome assembly (for this analysis, the output of the SOAPdenovo/GapCloser assembly of female RNA-seq reads was used without further processing other than selecting transcripts above 300 bps) using Bowtie2 with default parameters. Cufflinks was used to estimate FPKM values from the RNA-seq alignments, and male and female genomic read counts were obtained from the DNA-seq alignments for each transcript (after removal of alignments with mismatches). Transcripts with male over female read counts below 0.1, total female read counts above 30, and female-specific expression were classified as putative W-linked sequences. PCR primers were designed for 9 of these transcripts, and W-linkage confirmed when they yielded female-specific bands in a standard PCR (see Figure S11 for details on the PCR analysis).

### Estimation of K_a_, K_s_, and K_a_/K_s_

Coding sequences were extracted from the pygmy rattlesnake, garter snake and boa genome assemblies according to their homology to *Anolis* genes. Specifically, we mapped each *Anolis* CDS (see “Transcriptome Assembly”) to the snake genomes using blat with a translated query and database. In order to avoid the inclusion of paralogs, we used a reciprocal best hit approach: for each *Anolis* gene, only the genomic location with the largest mapping score was kept; similarly, when two *Anolis* genes mapped to the same snake genomic region (with an overlap longer than 20 bps), only the gene with the highest score was kept. These tables of reciprocal best hits were used to extract the putative gene loci from the genomic sequences of the three snake species with a perl script. Genewise was then run on these loci with default parameters, and the corresponding *Anolis* proteins as query, to retrieve the respective coding sequences. Only genes for which the resulting coding sequence encoded a protein longer than 40 amino acids with no stop codons in all three snake species were kept for further analysis.

In order to obtain alignments based on the protein sequences, we translated the coding sequences from all three snake species and aligned the translated peptide sequences, as well as the corresponding *Anolis* protein, with Muscle [68]. Gblocks [69] was run with default parameters to remove gaps and regions of low conservation from these protein alignments, resulting in shortened protein sequences of equal sizes in all the species. These proteins were then used as queries for Genewise to extract the corresponding DNA sequences from each of the snake and lizard species. Since the final DNA sequences from the snake and lizard species correspond exactly to their aligned protein sequences (after gap removal), they can simply be concatenated for each gene into one final DNA alignment (in a few cases Genewise yielded DNA sequences of different sizes for the different species; these were removed from the analysis. The final sample consists of 9553 genes). KaKs_calculator [70] was run with the Nei-Gojobori model to estimate pairwise K_a_ and K_s_ values for each gene between all species pairs (Dataset S2).

### Expression Analysis

Female and male reads were mapped back to the assembled transcriptome separately using Bowtie2 [71] with default parameters. FPKM values were estimated using Cufflinks [72] with a single GTF file per species that assigned a single transcript spanning the whole sequence to each gene (Dataset S1).

## Supporting Information

Dataset S1 **FPKM value and location in the** ***Anolis*** **genome of all transcripts analyzed in boa and pygmy rattlesnake, list of all genes (and location in the** ***Anolis*** **genome) detected in the genomes of boa, pygmy rattlesnake, and garter snake, and list of all W-candidate genes detected in pygmy rattlesnake, and garter snake.**
(XLSX)

Dataset S2 **Molecular evolution of Z-linked genes in snakes. K_a_, K_s_, and K_a_/K_s_ values for boa-** ***Anolis*** **, pygmy rattlesnake-** ***Anolis*** **and garter snake-** ***Anolis*** **comparisons are given for each gene.** All divergence values were obtained with the KaKs_calculator (http://code.google.com/p/kaks-calculator/) with the Nei-Gojobori model.
(XLSX)

Figure S1 **SNP count for each boa and pygmy rattlesnake gene along the genome.** Boa (A) and pygmy rattlesnake (B) genes were mapped according to their location in the *Anolis* genome, and, for each gene, the total number of SNPs was plotted. (C) The proportion of genes with SNPs for each macrochromosome.
(PDF)

Figure S2 **Comparative cytogenetic maps of sex chromosomes of snakes (picture redrawn from** [**9**] **) and** ***Anolis*** **(ideogram drawn for** ***Phython molorus*** **).** The location of 11 genes mapped in three snake species and their position along *Anolis* chromosome 6 is shown.
(PDF)

Figure S3 **Dot-plot between** ***Anolis*** **chromosomes and boa pseudochromosomes.** Boa Z scaffolds are ordered and oriented by finding the consensus order and orientation of blat hits between *Anolis* genes and boa scaffolds from Assemblathon 2 and tiling the sequences in the appropriate order and orientation. If the breakpoints of inversions or other structural rearrangements map within scaffolds, this will be seen as off-diagonal dots. Given the high-quality assembly of boa (the concatenated boa chromosome exhibits the following assembly statistics: N50 = 1,855 Kb; N90 = 574 Kb; and N95 = 345 Kb), we have high power for finding rearrangements present within euchromatin. This figure shows evidence for two inversions on the Z.
(PDF)

Figure S4 **Histogram of female-specific scaffolds mapping along the chromosomes of boa (A), garter snake (B), and pygmy rattlesnake (C).**
(PDF)

Figure S5 **Mapping of candidate female scaffolds to the genome of boa, garter snake, and pygmy rattlesnake with different stringency mapping parameters than in** **Figure 2** **.** (A) High stringency mapping, requiring that percent identity be above 30% and number of aligned nucleotides be at least 200. The W candidates homologous to Z-linked scaffolds make up 60% and 41% of all female-biased scaffolds in pygmy rattlesnake and garter snake genomes, respectively. The proportion of boa scaffolds (14%) does not differ significantly from random mapping (binomial test, *p* = 0.42). (B) Low stringency mapping, requiring only that number of aligned nucleotides be at least 100. The W candidates homologous to Z-linked scaffolds make up 50% and 34% of all female-biased scaffolds in pygmy rattlesnake and garter snake genomes, respectively. The proportion of boa scaffolds (6.8%) does not differ significantly from random mapping (binomial test, *p* = 0.72).
(PDF)

Figure S6 **Evolutionary strata and sequence conservation between the pygmy rattlesnake and garter snake W-candidate scaffolds mapped along the Z chromosome.** Legend like Figure 3, but shown are the data for the lower/higher stringency mapping as in Figure S5. (A) High stringency mapping, requiring that percent identity be above 30% and number of aligned nucleotides be at least 200. Median Z-W identity for the distal left and right strata are 55% and 56% for pygmy rattlesnake and 53% and 51% for garter snake, respectively. (B) Low stringency mapping, requiring only that number of aligned nucleotides be at least 100. Median Z-W identity for the distal left and right strata are 55% and 55% for pygmy rattlesnake and 51% and 48% for garter snake, respectively.
(PDF)

Figure S7 **Synonymous divergence between Z-W gametologs along the Z chromosome of garter snake and pygmy rattlesnake.** The grey line shows median synonymous divergence for autosomal loci, and the red line shows median divergence for Z-linked loci.
(PDF)

Figure S8 **Comparison of location of female-specific scaffolds in pygmy rattlesnake (top) and garter snakes (bottom).** Regions of overlap are indicated in the middle. The mapping following Figures 2 and 3 are shown.
(PDF)

Figure S9 **Gene conservation on the W of garter snake and pygmy rattlesnake.** *Anolis* chromosome 6 (Z of snakes) was divided into three bins of equal sizes (45589–26937164, 26937164–53828738, 53828738–80720312, where 45589 and 80720312 are the location of the first and last gene along the chromosome). For each bin, the total number of genes was compared to the number of genes detected on putative W-linked scaffolds of garter snake, pygmy rattlesnake, or of both species (A). The number of genes detected on putative W-linked scaffolds of both species is higher than expected randomly (15 in total versus 1.9 expected, *p*<0.001 with a goodness of fit Chi-square test, where the expected proportion of overlapping genes in simply the proportion of chromosome 6/Z genes found on the pygmy W times the proportion of chromosome 6/Z genes found on the garter snake W) for all intervals (B).
(PDF)

Figure S10 **Gene trees of Z and W gametologs from pygmy rattlesnake and garter snake.** For some genes, we also added outgroup sequence information from *Anolis* or boa.
(PDF)

Figure S11 **PCR confirmation of W-linkage of six female-specific transcripts in pygmy rattlesnake.** Primers were designed to amplify fragments of putative W-linked scaffolds, while primers designed to amplify fragments of two autosomal sequences were used as a control. For each set of primers, standard PCR was performed with either male or female DNA as template (from the same samples used for the genomic sequencing), and an annealing temperature of 58°C. W-linkage was confirmed by the appearance of female-specific bands.
(PDF)

Figure S12 **Log_2_ of expression in female and male, and log_2_ of female over male expression, for the different macrochromosomes of pygmy rattlesnake using different cutoff values.** Only genes with FPKM values over 0 (upper panel), 1 (middle panel), or 10 (lower panel) were used in the analysis.
(PDF)

Figure S13 **Synteny of genes between** ***Anolis*** **chromosomes and the eight largest boa scaffolds that mapped to macrochromosomes.** *Anolis* CDS sequences were mapped to the boa genomic scaffolds using blat. The corresponding location of each gene on the eight largest scaffolds that mapped to *Anolis* macrochromosomes was plotted against their location on the corresponding *Anolis* chromosome.
(PDF)

Figure S14 **Location of all 431 Z-linked pygmy rattlesnake transcripts (grey) and of the 80 genes with log_2_(F/M expression) = 0±0.3 (red, corresponds approximately to 0.8<F/M<1.2) along chromosome 6 (Z).**
(PDF)

Table S1 **Assembly statistics for boa, pygmy rattlesnake, and garter snake genomes.**
(DOCX)

Table S2 **Mapping of known Z-linked and autosomal markers of rat snake to the** ***Anolis*** **genome.**
(DOCX)

Table S3 **Average pairwise synonymous and non-synonymous divergence between all the species used in this study.**
(DOCX)

Table S4 **Divergence at synonymous and nonsynonymous sites between Z-W gametologs.**
(DOCX)

Table S5 **Synonymous divergence K_s_ between ZW paralogs using three other models to estimate K_s_.**
(DOCX)

Table S6 **List of confirmed and putative W-linked transcripts in pygmy rattlesnake.** Female and male RNA-seq and DNA-seq reads were mapped to the *de novo* transcriptome using Bowtie2, and the resulting alignments were used to estimate expression levels (FPKM, with Cufflinks) and genomic male and female read counts. Transcripts with male/female read counts below 0.1 and female-specific expression were classified as putative W-derived sequences (only transcripts with at least 30 genomic female reads were considered in this analysis). The PCR confirmation of W-linkage of some of the transcripts is described in Figure S11.
(DOCX)

Table S7 **Detecting male-driven and faster-Z evolution using three other models to estimate K_a_ and K_s_.** Median K_a_, K_s_, and K_a_/K_s_ values for boa-*Anolis*, pygmy rattlesnake-*Anolis* and garter snake-*Anolis* comparisons are given for autosomal macrochromosomes and for chromosome 6/Z. All estimates of rates of evolution were obtained using KaKs_calculator.
(DOCX)

Table S8 **Number of RNA-seq and DNA-seq reads generated for/used in this analysis.**
(DOCX)

## Abbreviations

FPKM: fragments per kilobase of transcript per million mapped reads
PAR: pseudoautosomal region
