# Supplementary figures and images for: Comparative Sex Chromosome Genomics in Snakes: Differentiation, Evolutionary Strata, and Lack of Global Dosage Compensation
Source: PLoS Biol. 2013 Aug 27;11(8):e1001643. doi: 10.1371/journal.pbio.1001643 (PMC3754893; doi:10.1371/journal.pbio.1001643)

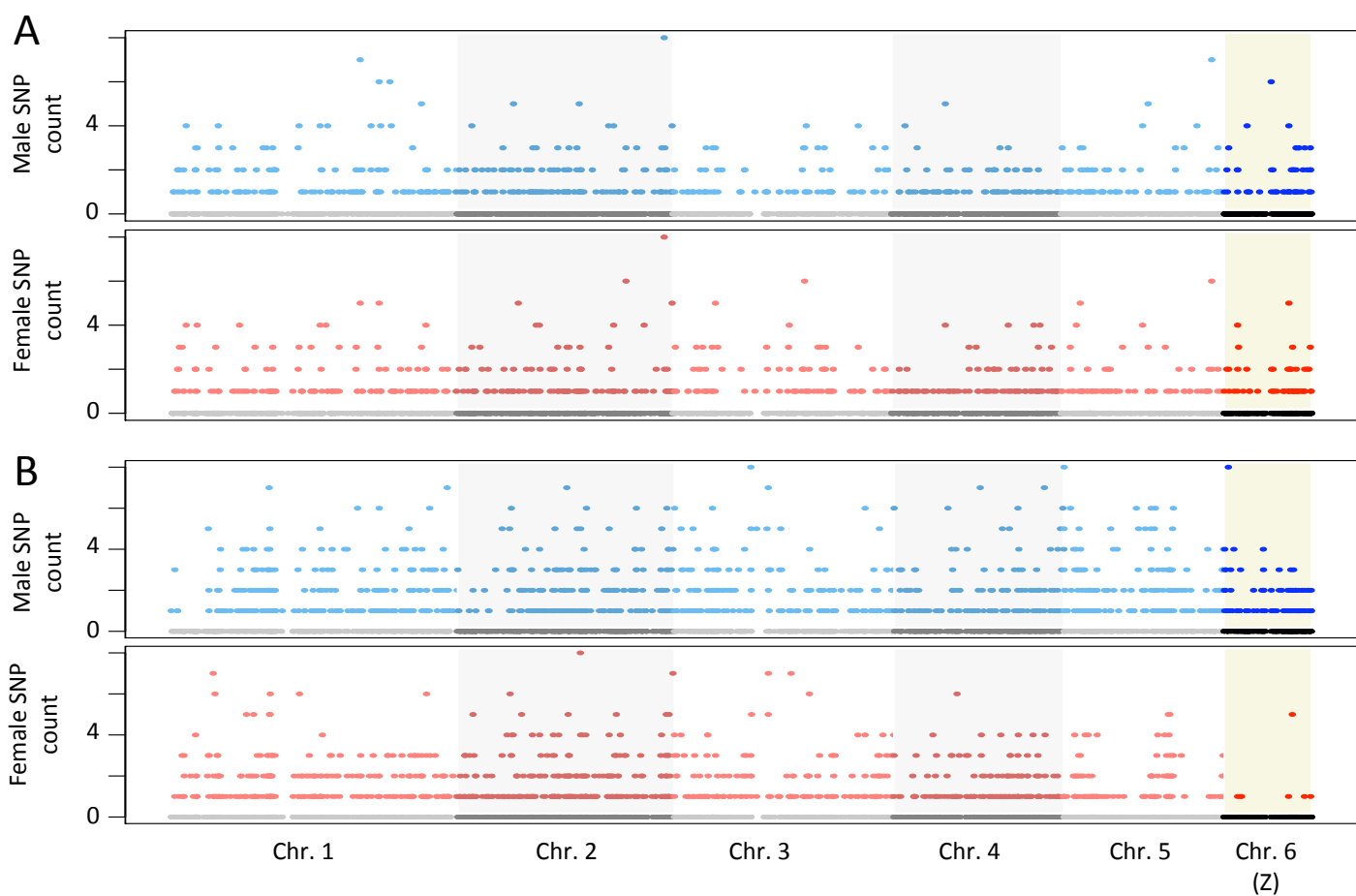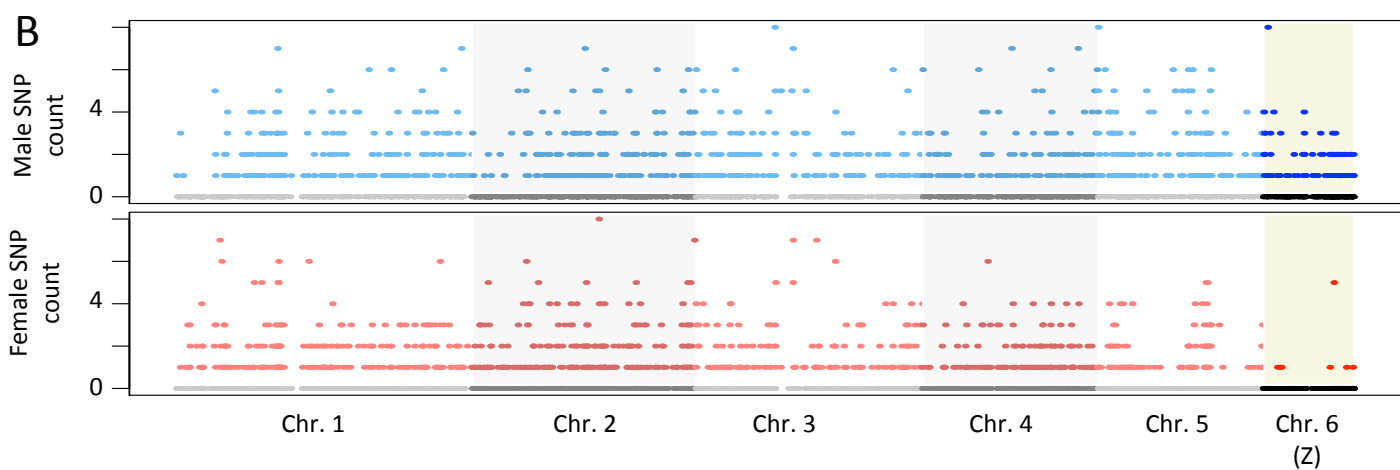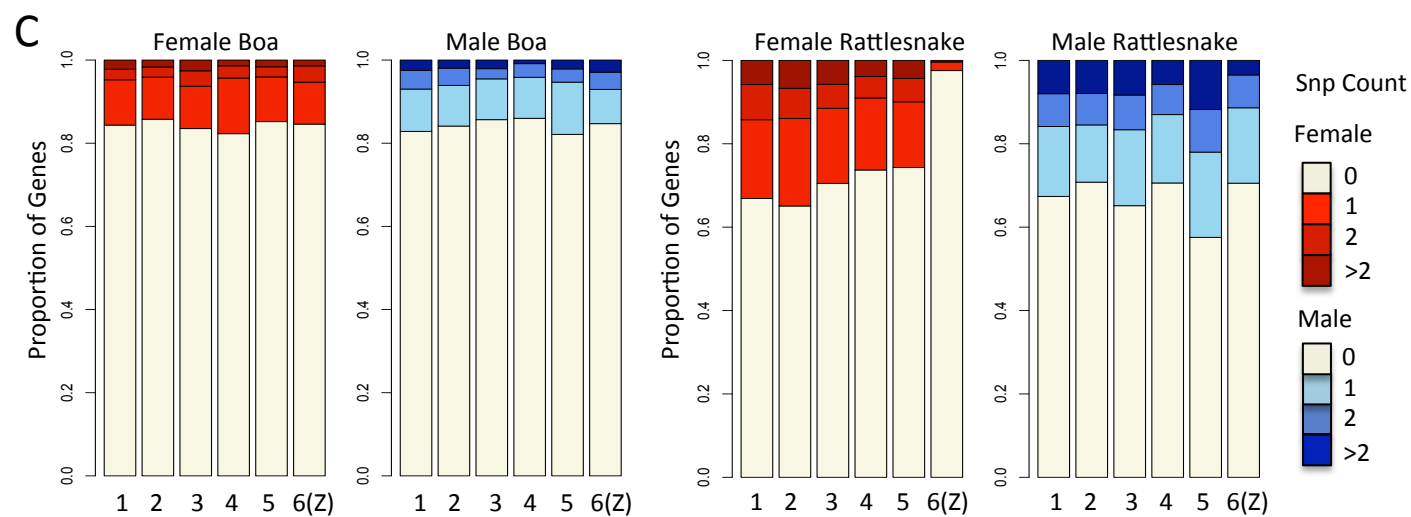

Supplement: Figure S1 — SNP count for each boa and pygmy rattlesnake gene along the genome. Boa (A) and pygmy rattlesnake (B) genes were mapped according to their location in the Anolis genome, and, for each gene, the total number of SNPs was plotted. (C) The proportion of genes with SNPs for each macrochromosome. (PDF) [file pbio.1001643.s003.pdf]

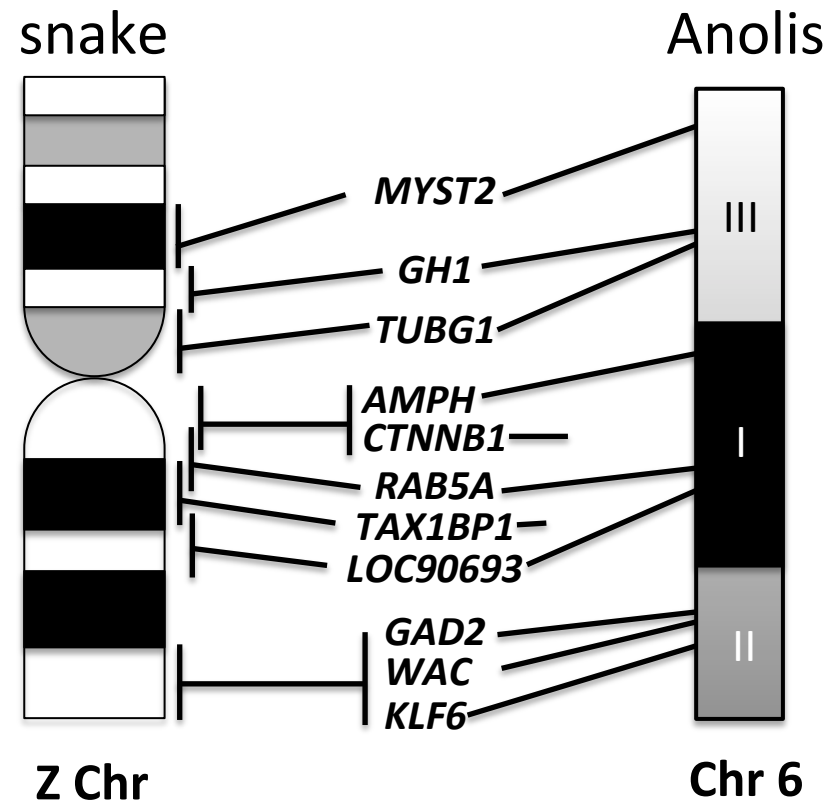

Supplement: Figure S2 — Comparative cytogenetic maps of sex chromosomes of snakes (picture redrawn from [9] ) and Anolis (ideogram drawn for Phython molorus ). The location of 11 genes mapped in three snake species and their position along Anolis chromosome 6 is shown. (PDF) [file pbio.1001643.s004.pdf]

chromosome 1

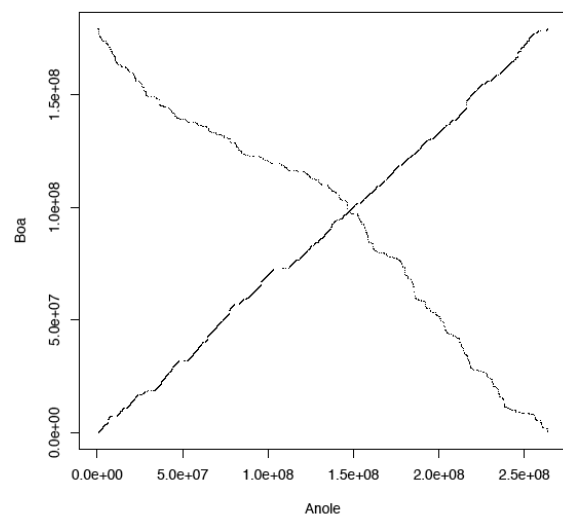

chromosome 2

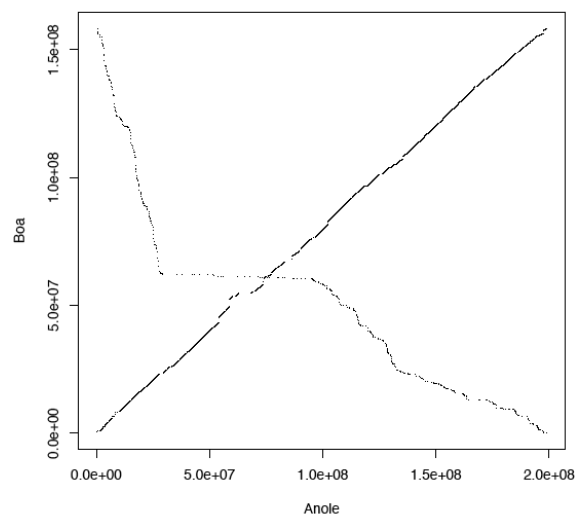

chromosome 3

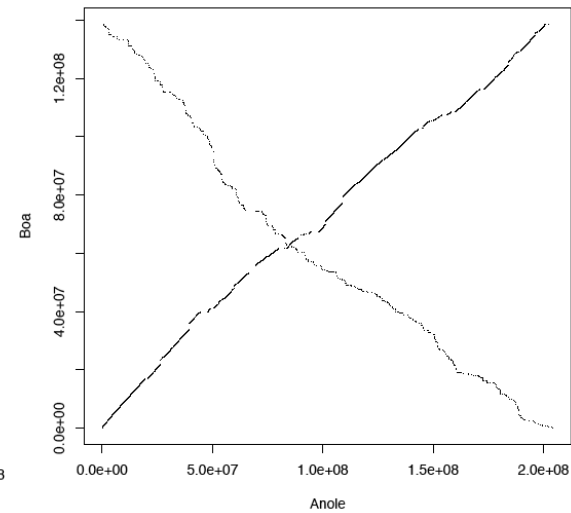

chromosome 4

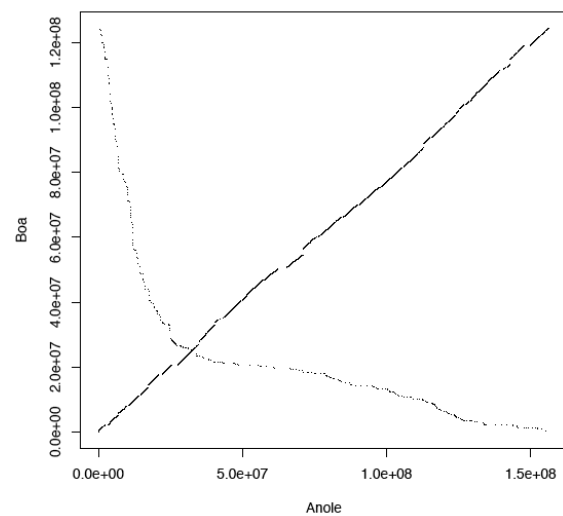

chromosome 5

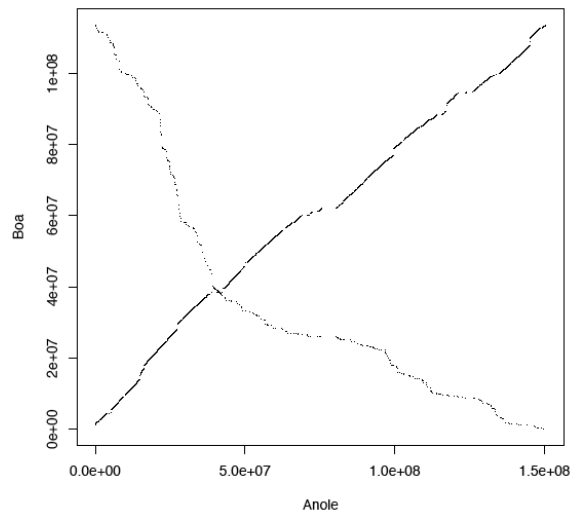

chromosome 6

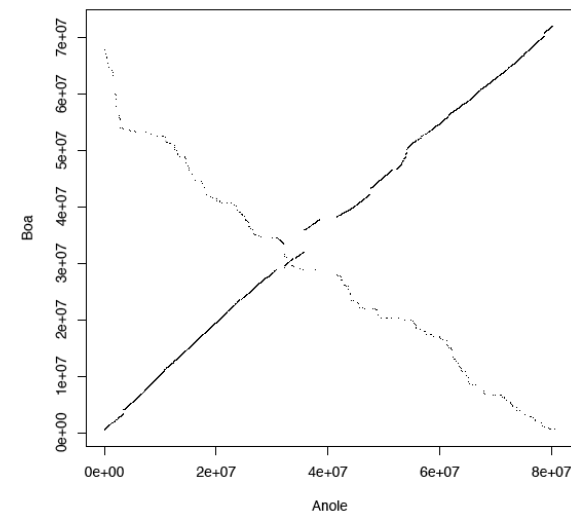

Supplement: Figure S3 — Dot-plot between Anolis chromosomes and boa pseudochromosomes. Boa Z scaffolds are ordered and oriented by finding the consensus order and orientation of blat hits between Anolis genes and boa scaffolds from Assemblathon 2 and tiling the sequences in the appropriate order and orientation. If the breakpoints of inversions or other structural rearrangements map within scaffolds, this will be seen as off-diagonal dots. Given the high-quality assembly of boa (the concatenated boa chromosome exhibits the following assembly statistics: N50 = 1,855 Kb; N90 = 574 Kb; and N95 = 345 Kb), we have high power for finding rearrangements present within euchromatin. This figure shows evidence for two inversions on the Z. (PDF) [file pbio.1001643.s005.pdf]

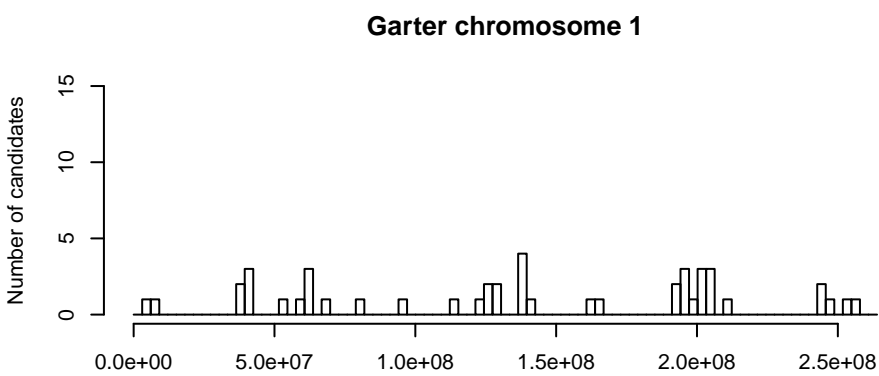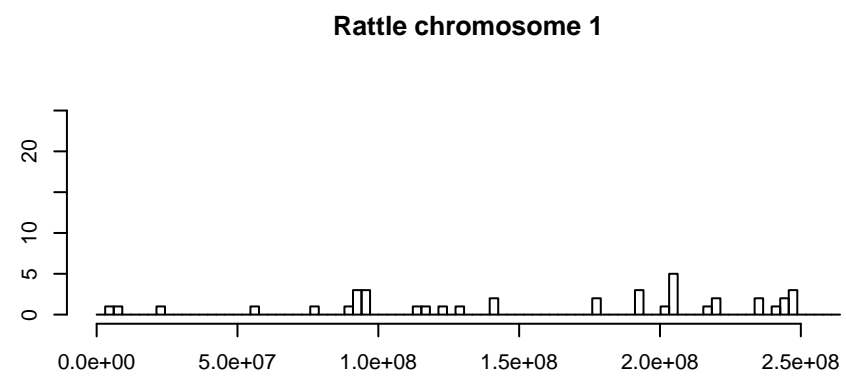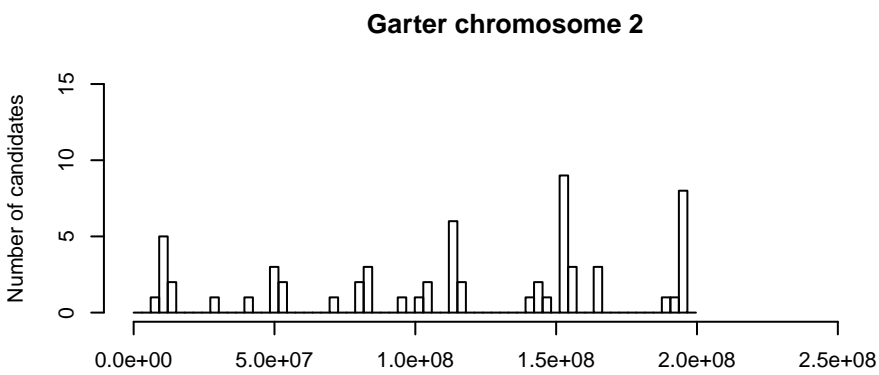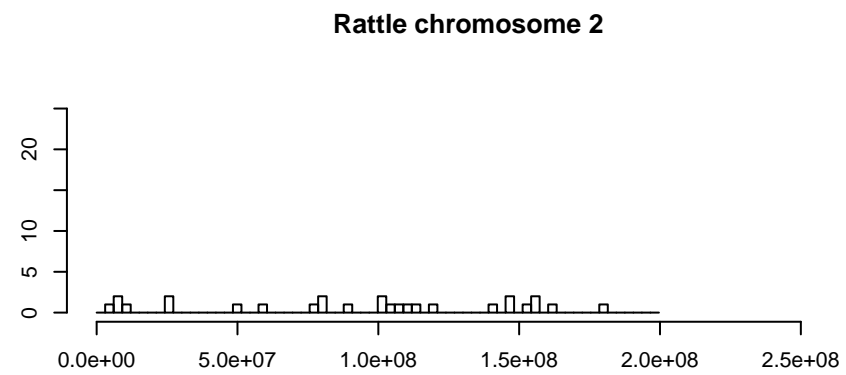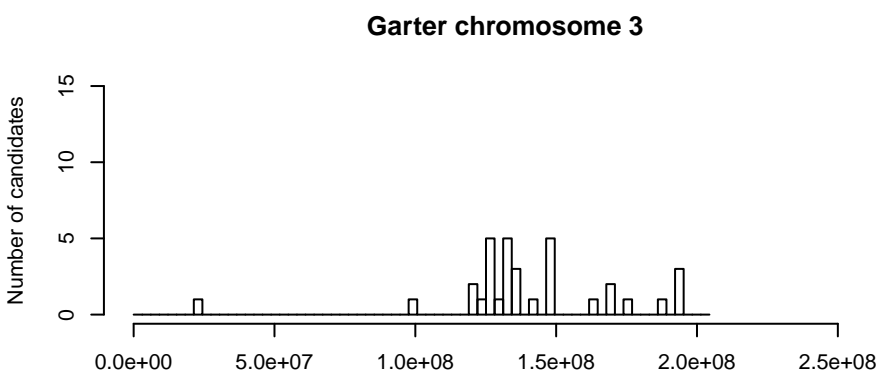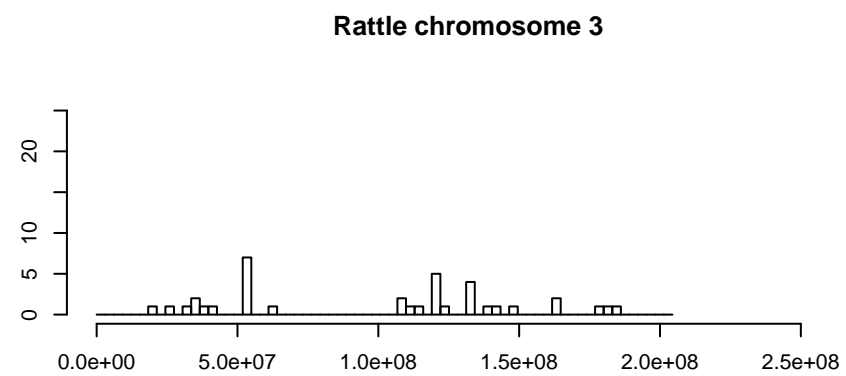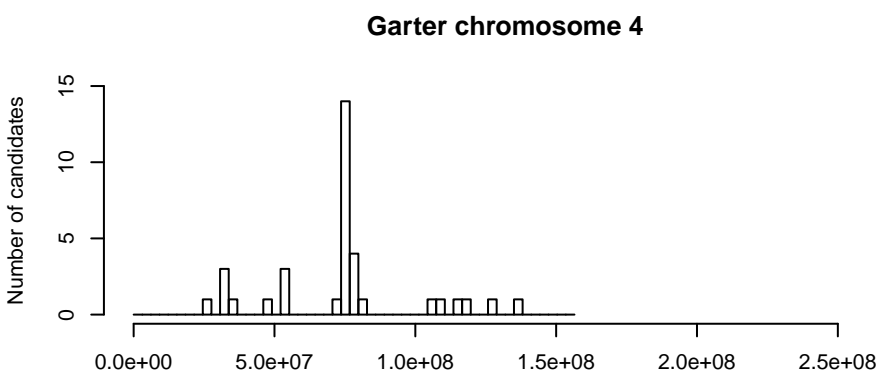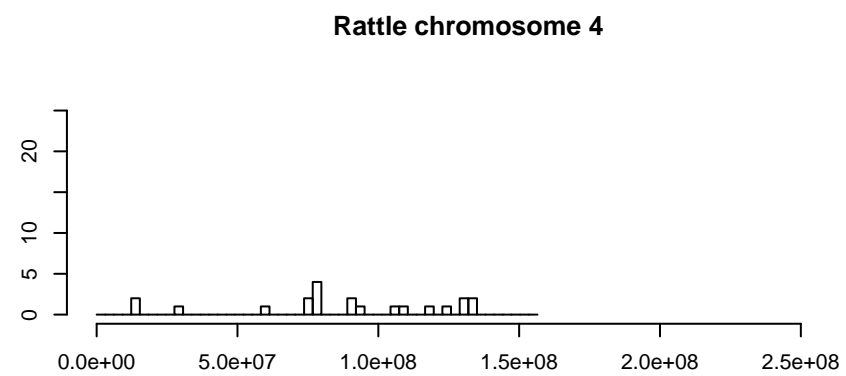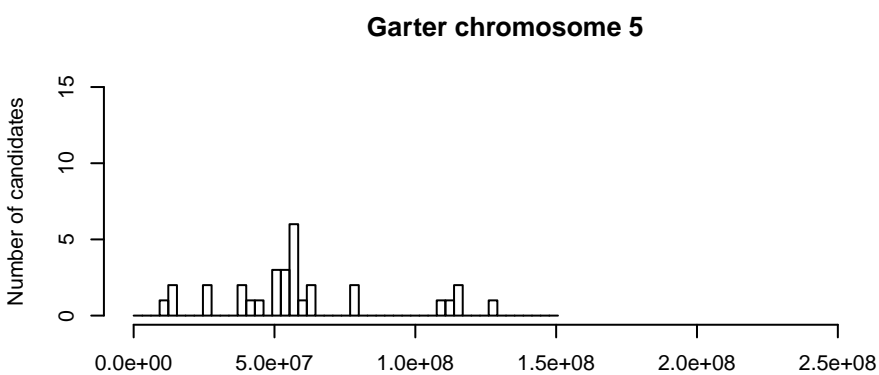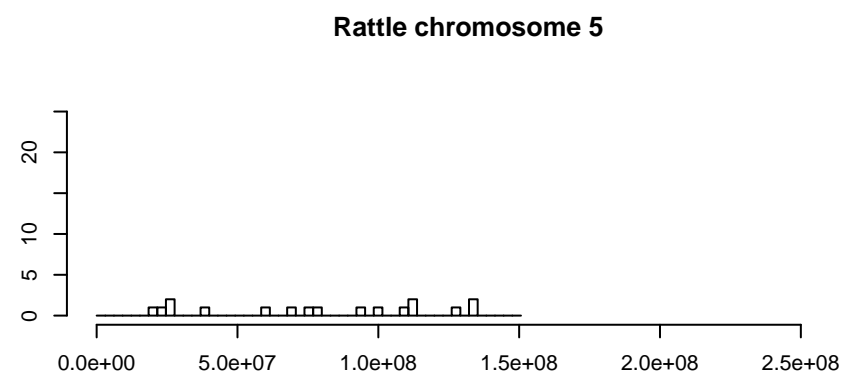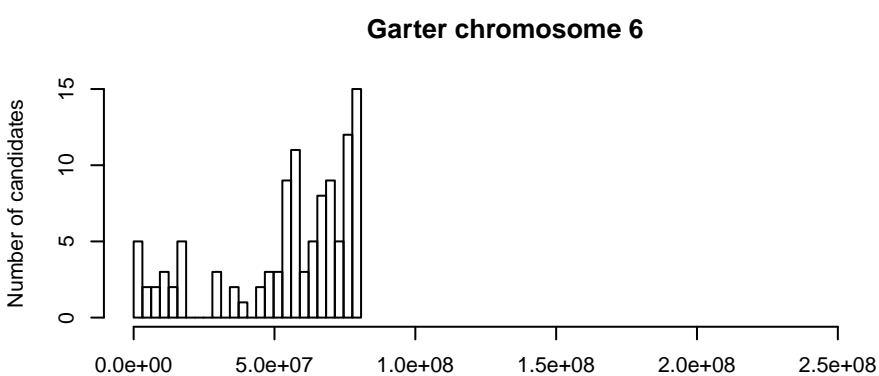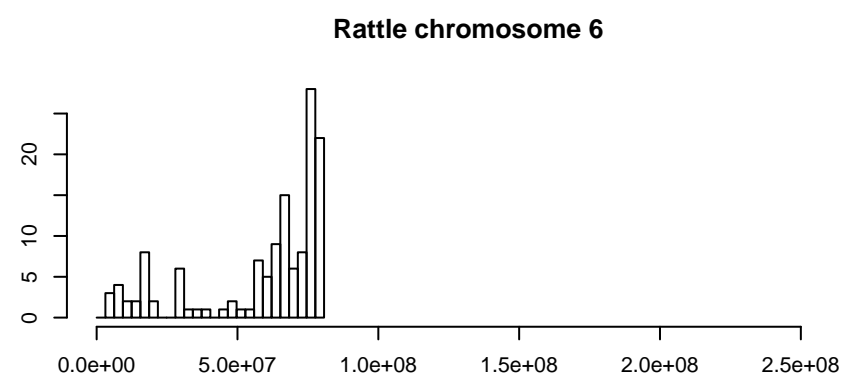

Supplement: Figure S4 — Histogram of female-specific scaffolds mapping along the chromosomes of boa (A), garter snake (B), and pygmy rattlesnake (C). (PDF) [file pbio.1001643.s006.pdf]

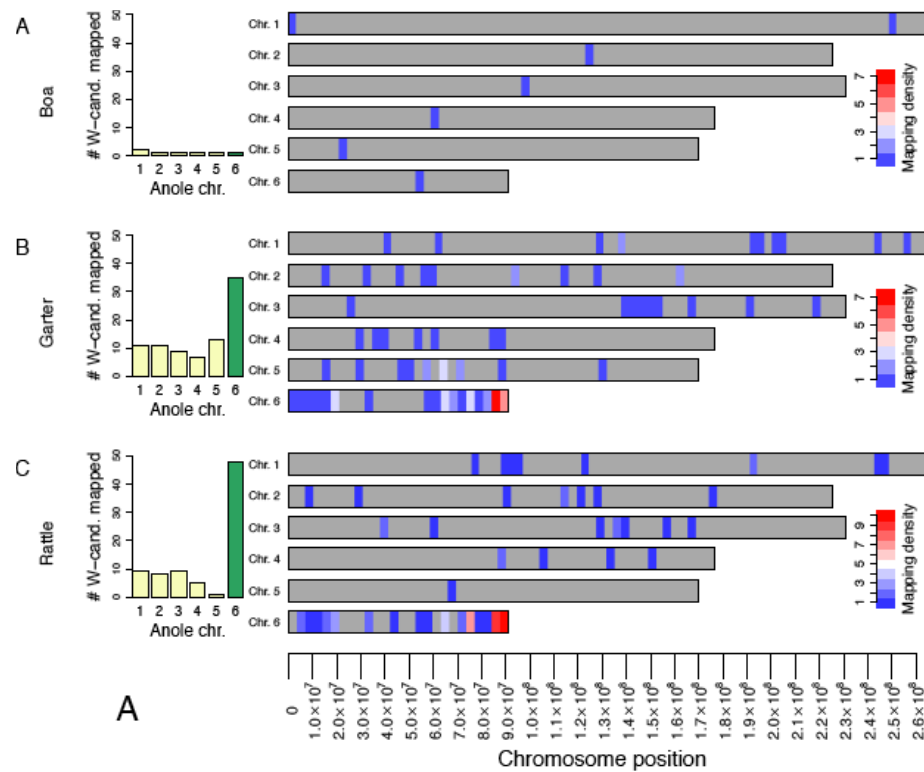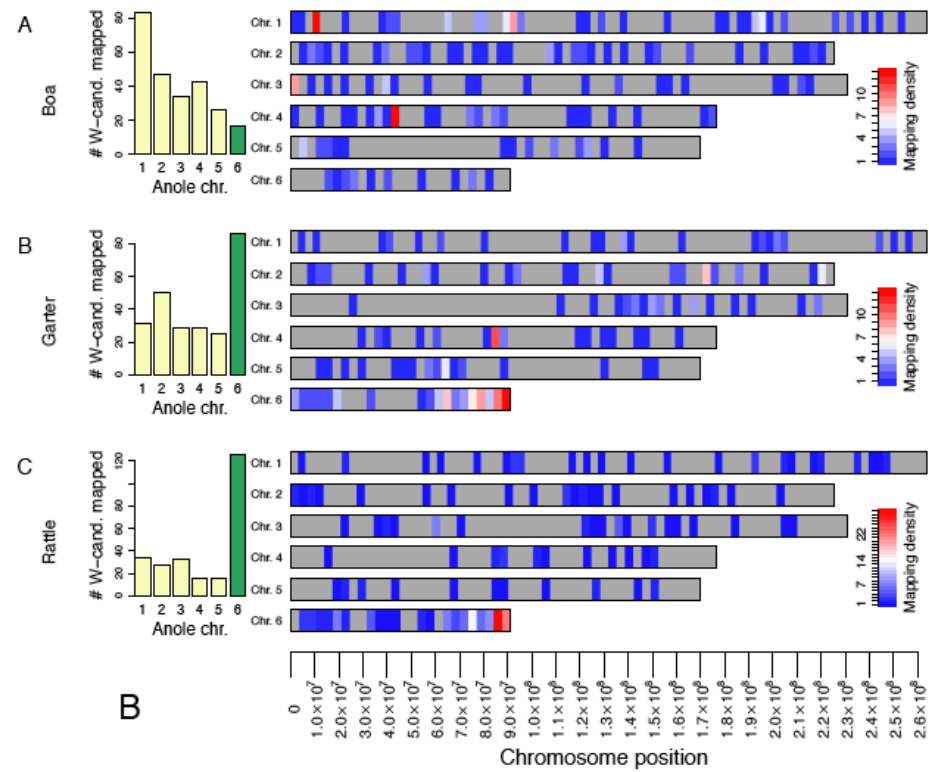

Figure S5

Supplement: Figure S5 — Mapping of candidate female scaffolds to the genome of boa, garter snake, and pygmy rattlesnake with different stringency mapping parameters than in Figure 2 . (A) High stringency mapping, requiring that percent identity be above 30% and number of aligned nucleotides be at least 200. The W candidates homologous to Z-linked scaffolds make up 60% and 41% of all female-biased scaffolds in pygmy rattlesnake and garter snake genomes, respectively. The proportion of boa scaffolds (14%) does not differ significantly from random mapping (binomial test, p = 0.42). (B) Low stringency mapping, requiring only that number of aligned nucleotides be at least 100. The W candidates homologous to Z-linked scaffolds make up 50% and 34% of all female-biased scaffolds in pygmy rattlesnake and garter snake genomes, respectively. The proportion of boa scaffolds (6.8%) does not differ significantly from random mapping (binomial test, p = 0.72). (PDF) [file pbio.1001643.s007.pdf]

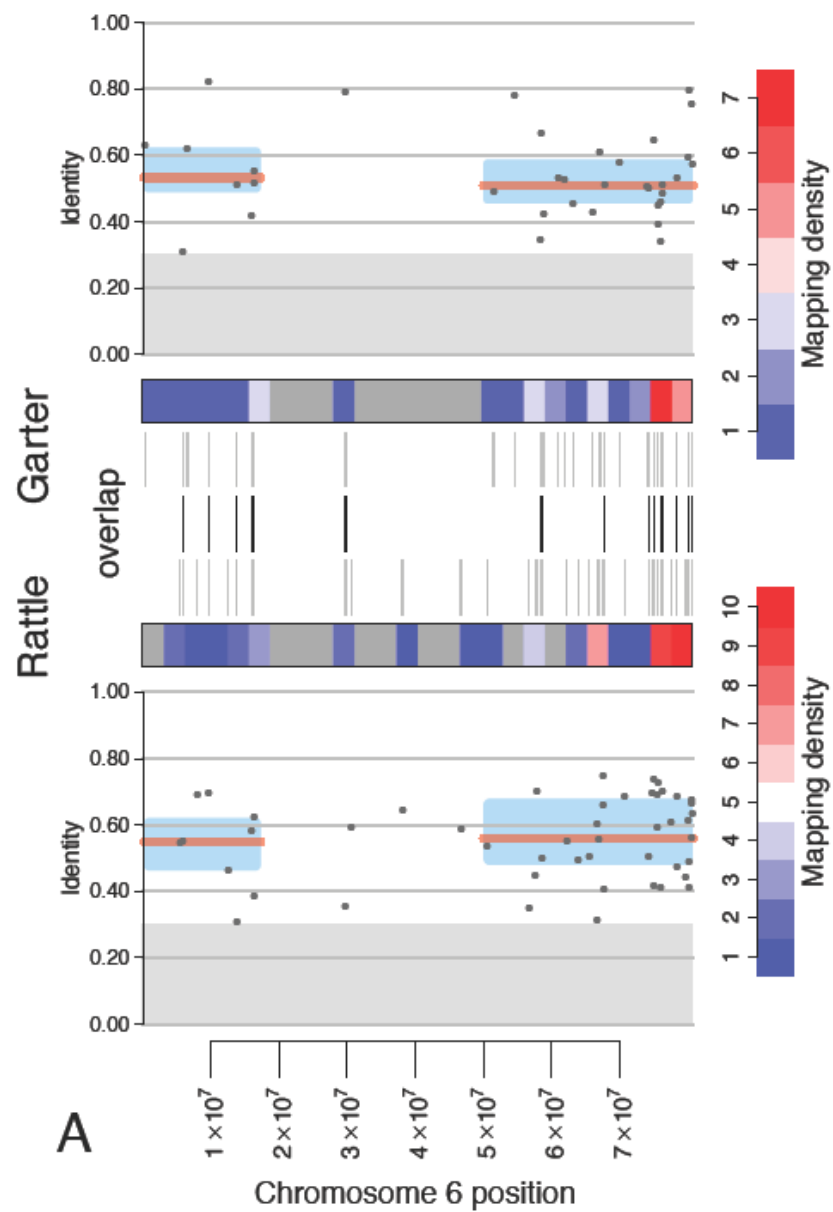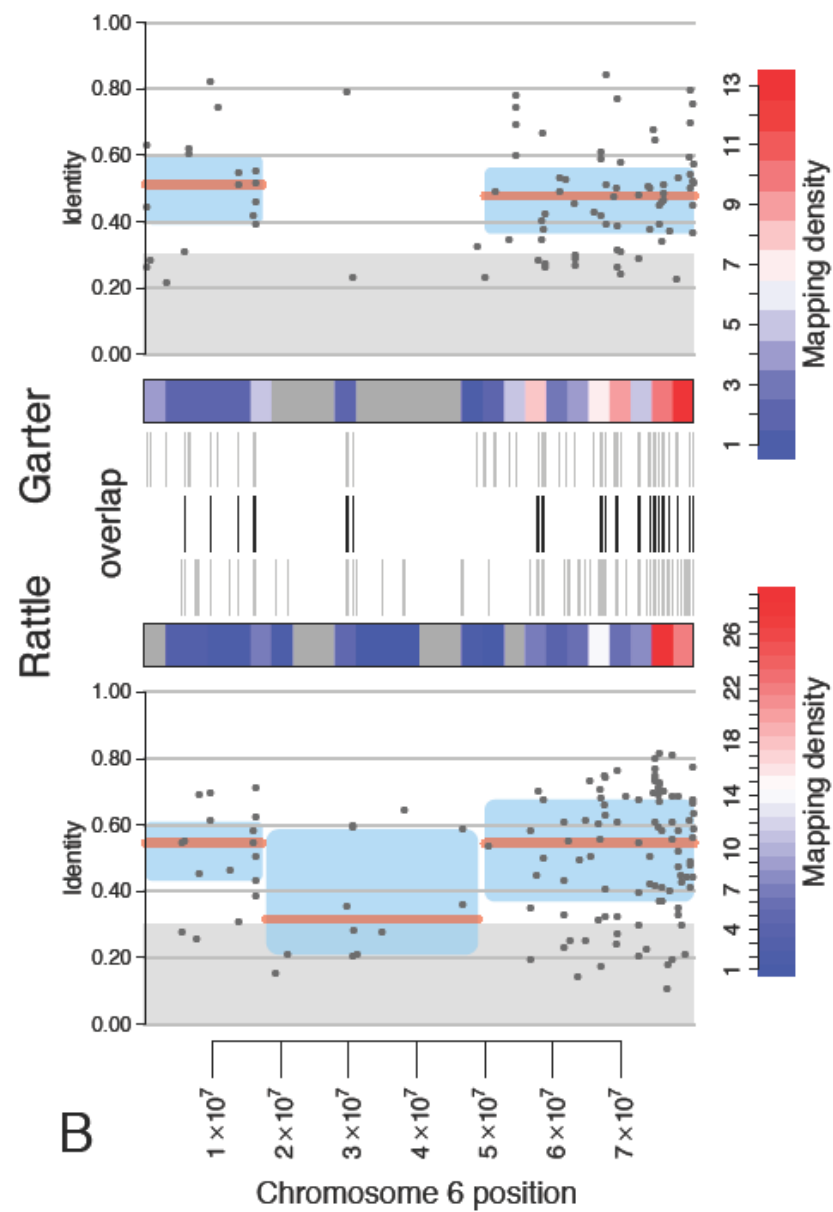

Figure S6

Supplement: Figure S6 — Evolutionary strata and sequence conservation between the pygmy rattlesnake and garter snake W-candidate scaffolds mapped along the Z chromosome. Legend like Figure 3, but shown are the data for the lower/higher stringency mapping as in Figure S5. (A) High stringency mapping, requiring that percent identity be above 30% and number of aligned nucleotides be at least 200. Median Z-W identity for the distal left and right strata are 55% and 56% for pygmy rattlesnake and 53% and 51% for garter snake, respectively. (B) Low stringency mapping, requiring only that number of aligned nucleotides be at least 100. Median Z-W identity for the distal left and right strata are 55% and 55% for pygmy rattlesnake and 51% and 48% for garter snake, respectively. (PDF) [file pbio.1001643.s008.pdf]

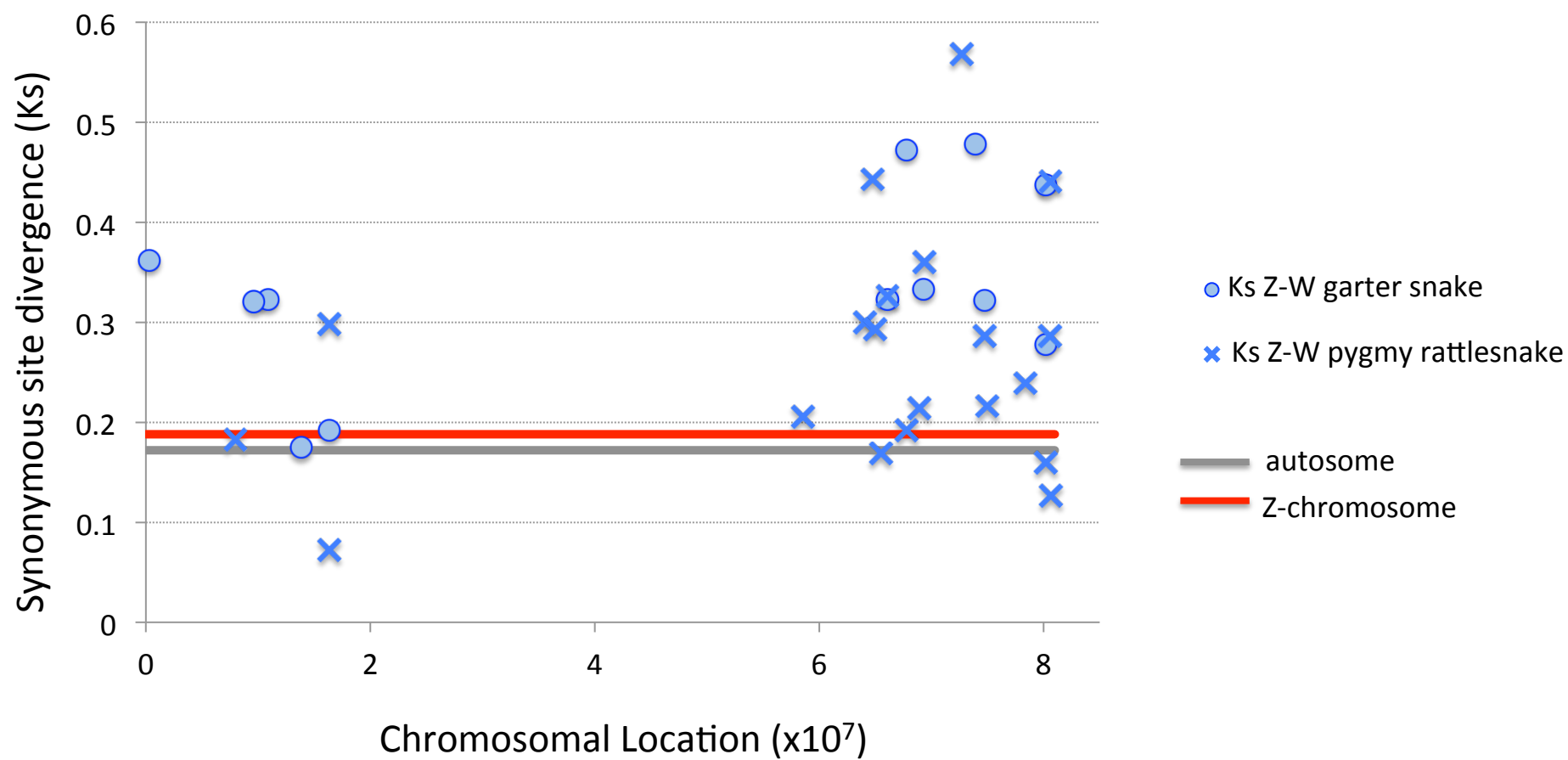

Supplement: Figure S7 — Synonymous divergence between Z-W gametologs along the Z chromosome of garter snake and pygmy rattlesnake. The grey line shows median synonymous divergence for autosomal loci, and the red line shows median divergence for Z-linked loci. (PDF) [file pbio.1001643.s009.pdf]

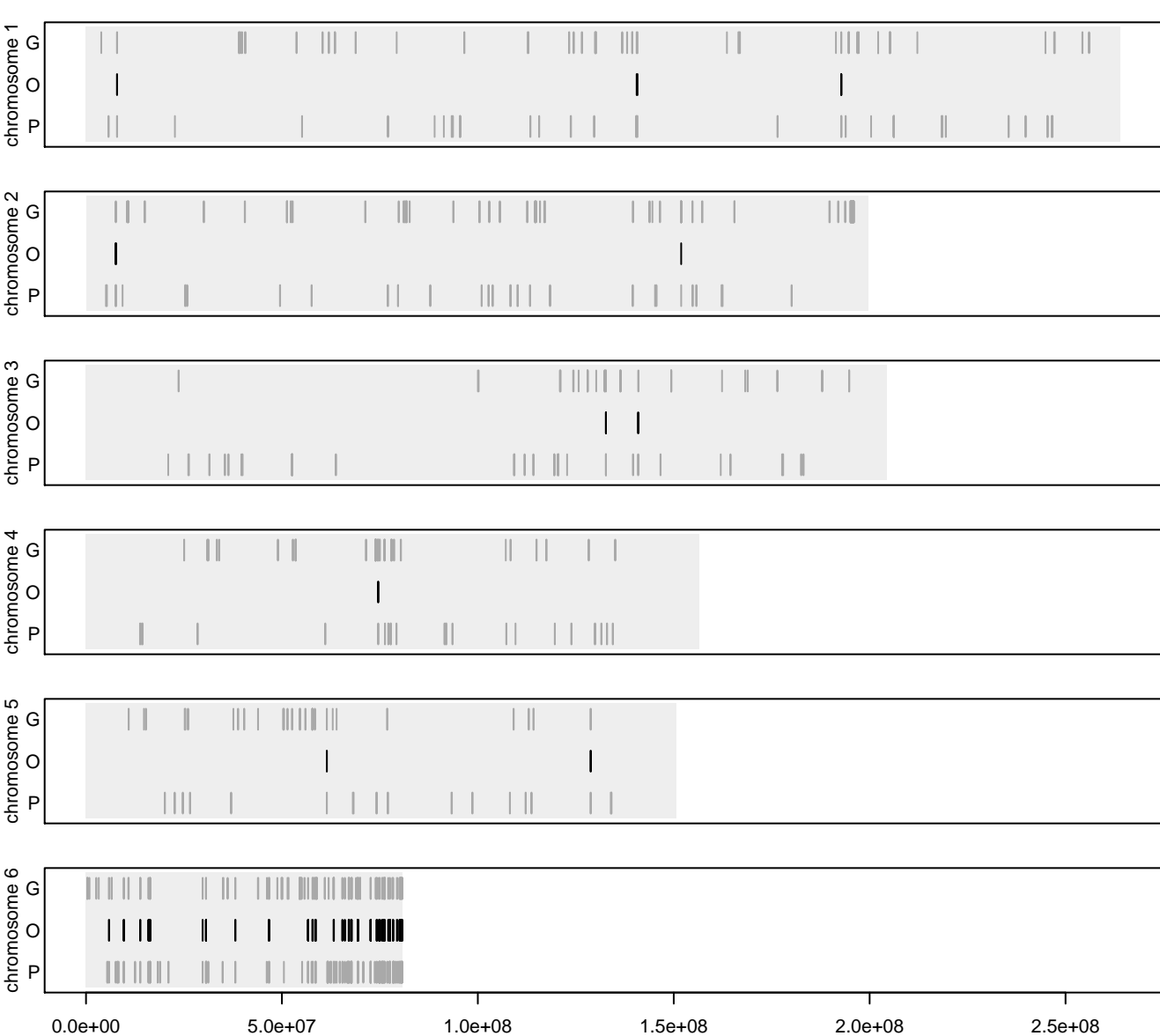

Chromosome Position

Supplement: Figure S8 — Comparison of location of female-specific scaffolds in pygmy rattlesnake (top) and garter snakes (bottom). Regions of overlap are indicated in the middle. The mapping following Figures 2 and 3 are shown. (PDF) [file pbio.1001643.s010.pdf]

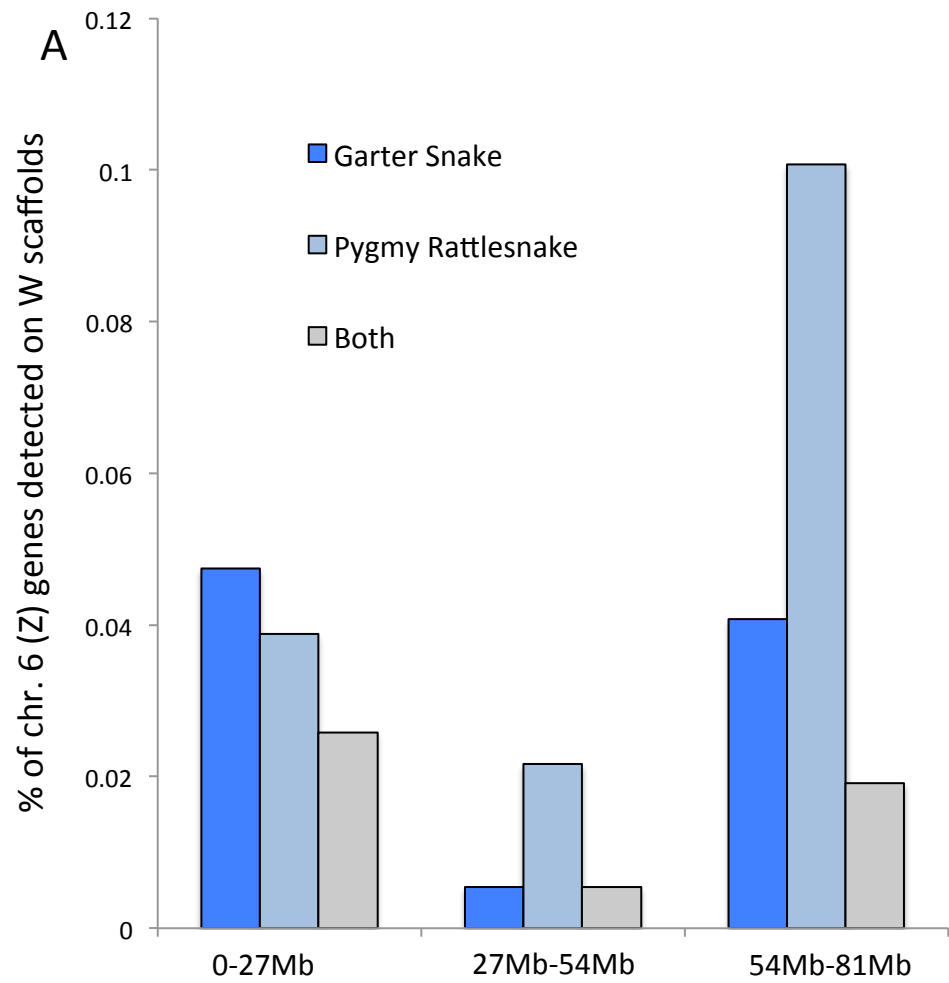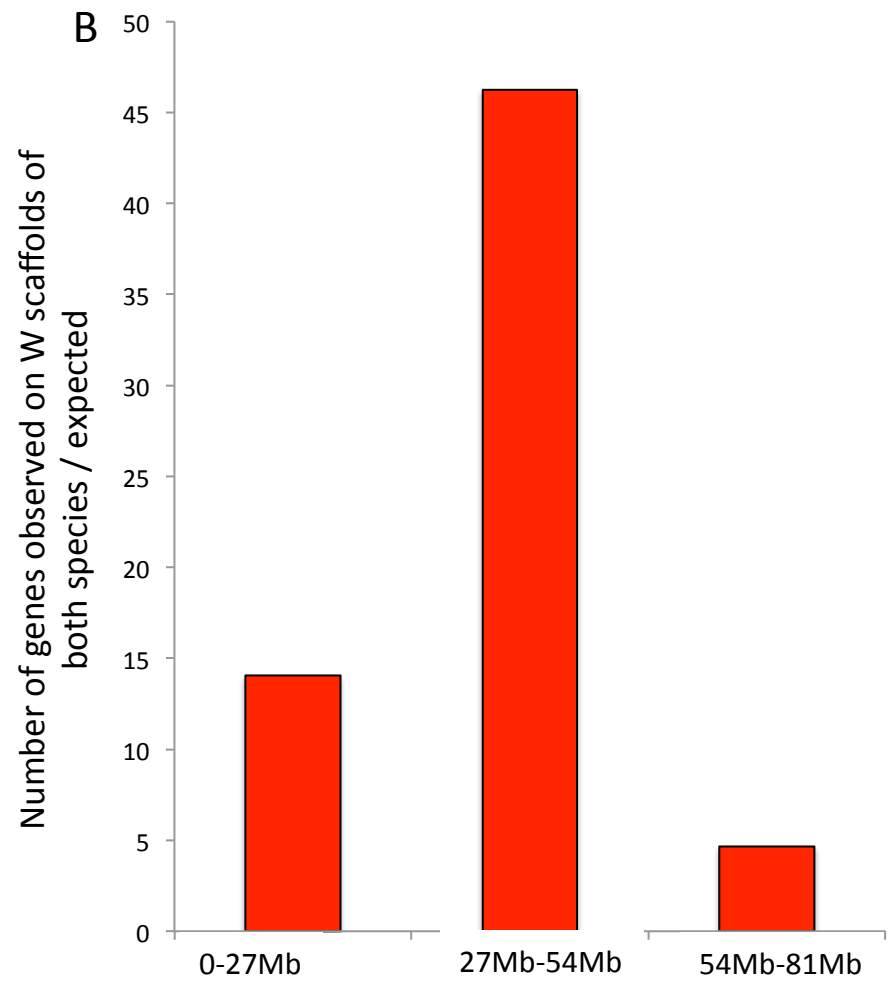

Supplement: Figure S9 — Gene conservation on the W of garter snake and pygmy rattlesnake. Anolis chromosome 6 (Z of snakes) was divided into three bins of equal sizes (45589–26937164, 26937164–53828738, 53828738–80720312, where 45589 and 80720312 are the location of the first and last gene along the chromosome). For each bin, the total number of genes was compared to the number of genes detected on putative W-linked scaffolds of garter snake, pygmy rattlesnake, or of both species (A). The number of genes detected on putative W-linked scaffolds of both species is higher than expected randomly (15 in total versus 1.9 expected, p<0.001 with a goodness of fit Chi-square test, where the expected proportion of overlapping genes in simply the proportion of chromosome 6/Z genes found on the pygmy W times the proportion of chromosome 6/Z genes found on the garter snake W) for all intervals (B). (PDF) [file pbio.1001643.s011.pdf]

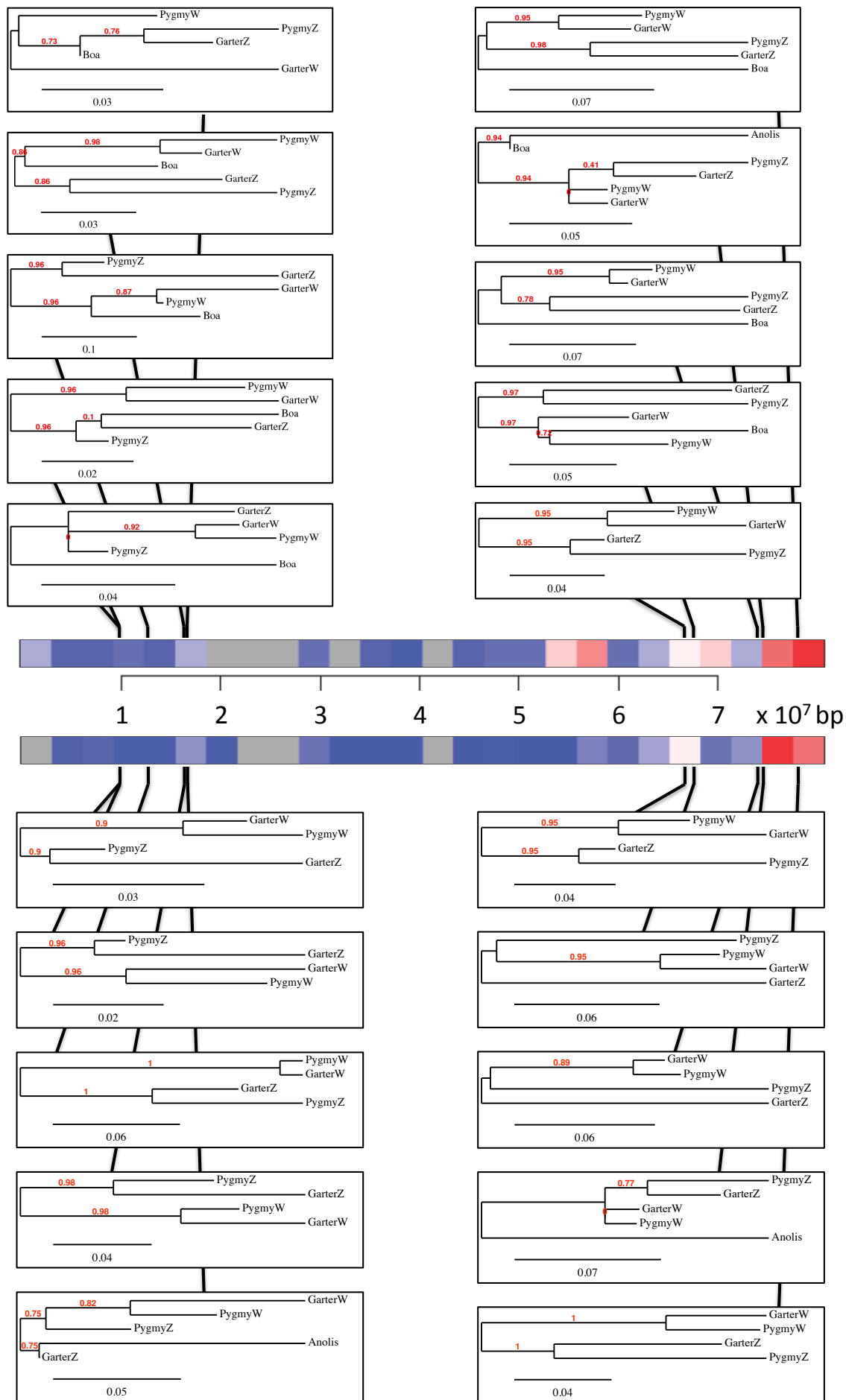

Supplement: Figure S10 — Gene trees of Z and W gametologs from pygmy rattlesnake and garter snake. For some genes, we also added outgroup sequence information from Anolis or boa. (PDF) [file pbio.1001643.s012.pdf]

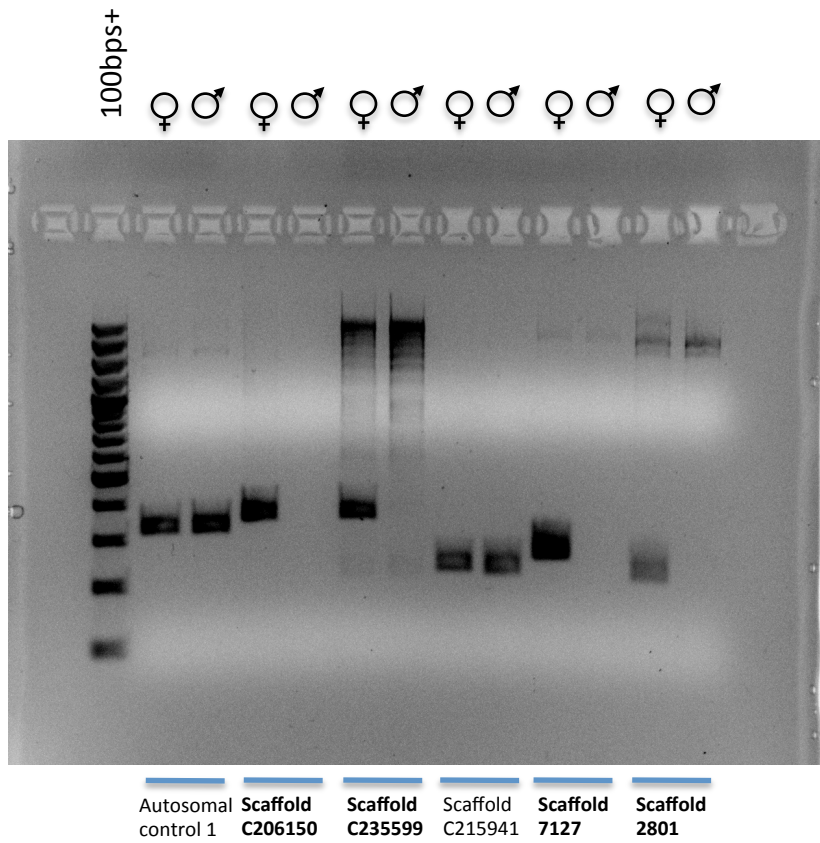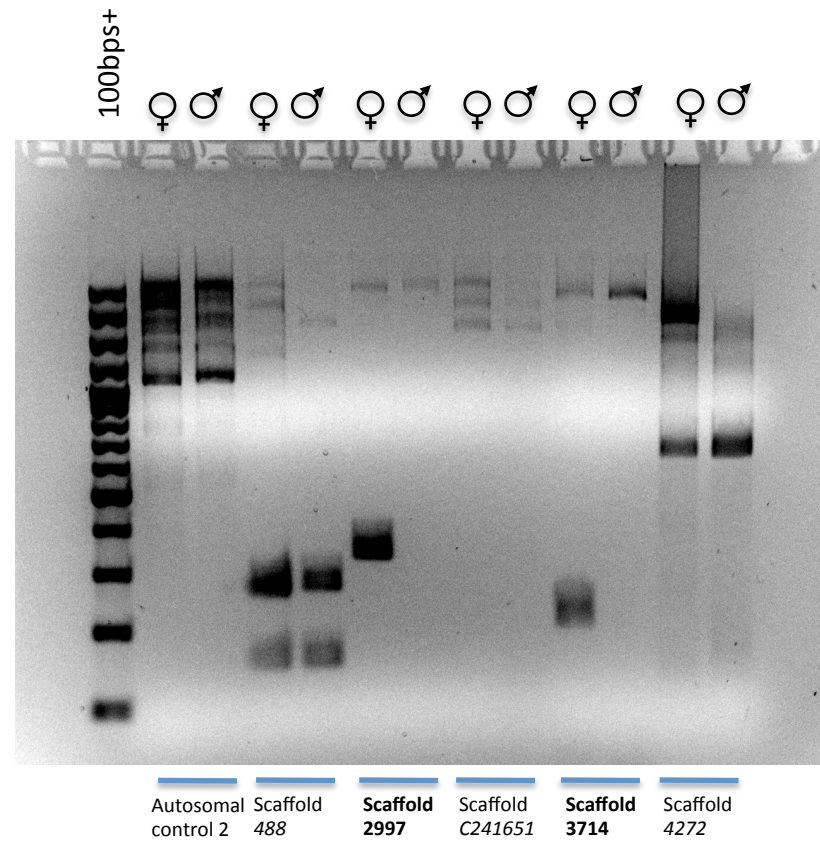

Supplement: Figure S11 — PCR confirmation of W-linkage of six female-specific transcripts in pygmy rattlesnake. Primers were designed to amplify fragments of putative W-linked scaffolds, while primers designed to amplify fragments of two autosomal sequences were used as a control. For each set of primers, standard PCR was performed with either male or female DNA as template (from the same samples used for the genomic sequencing), and an annealing temperature of 58°C. W-linkage was confirmed by the appearance of female-specific bands. (PDF) [file pbio.1001643.s013.pdf]

**All Genes**

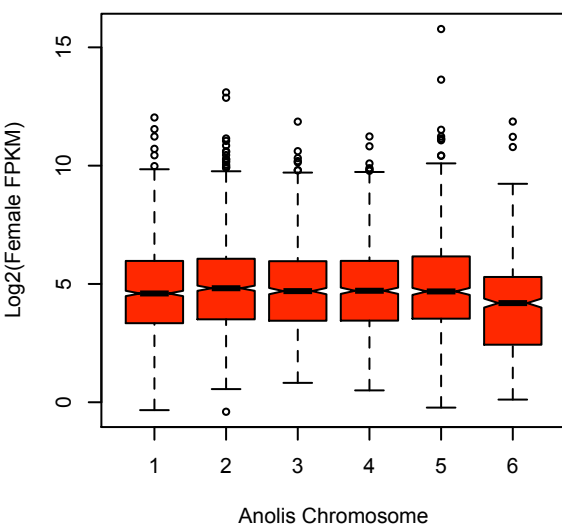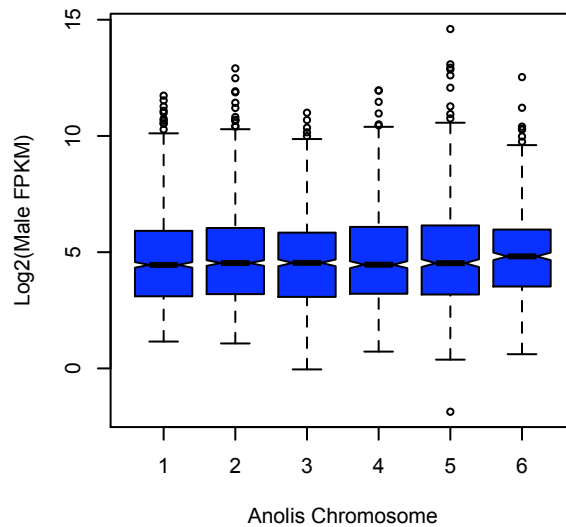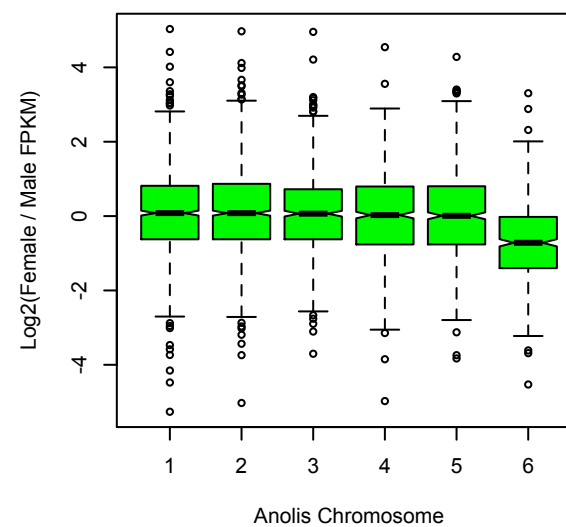

**Genes with FPKM>1**

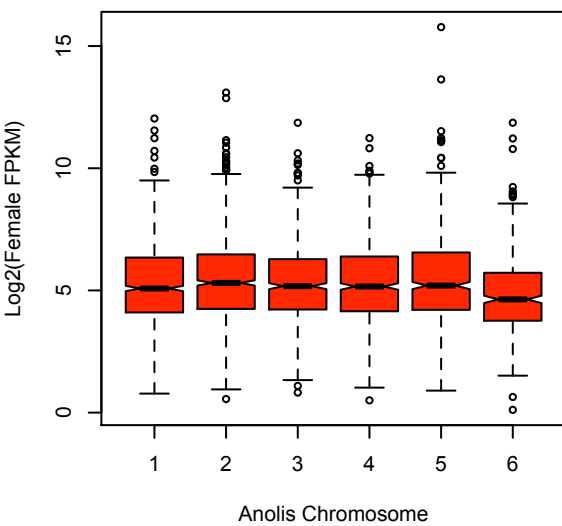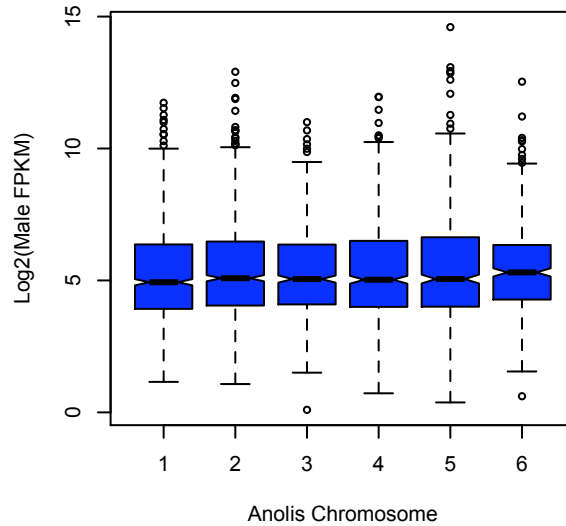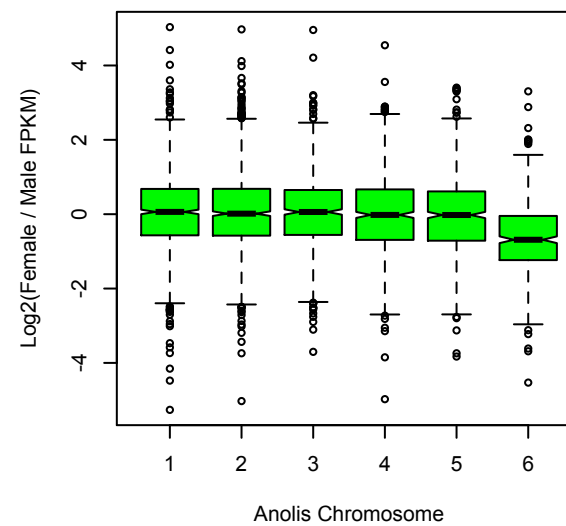

**Genes with FPKM>10**

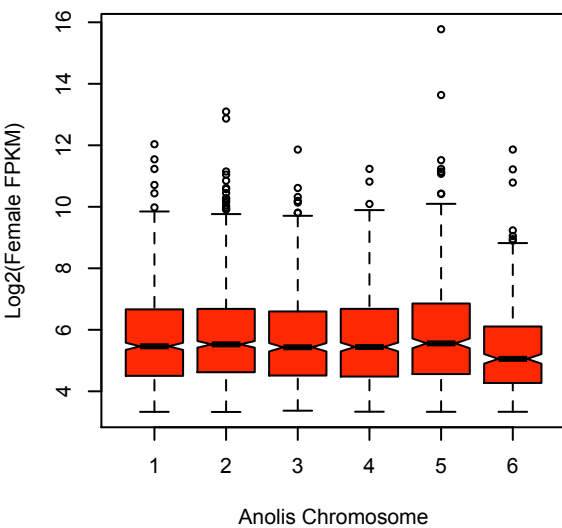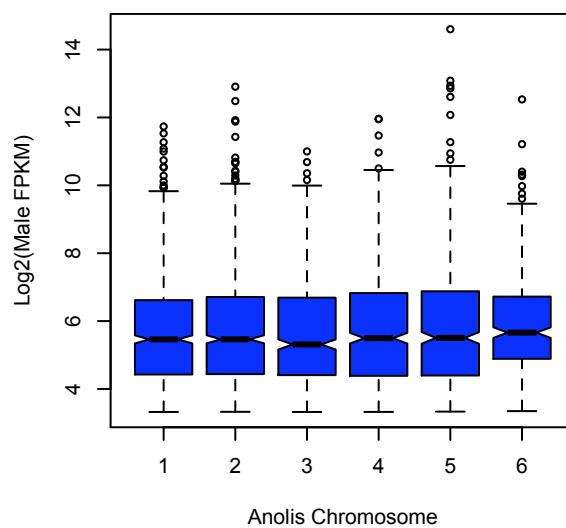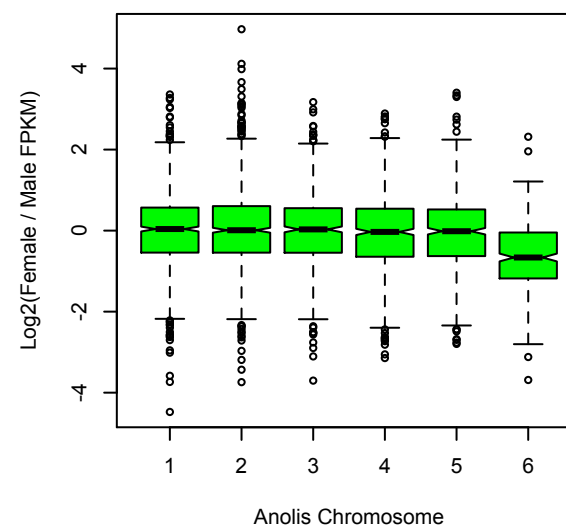

Supplement: Figure S12 — Log2 of expression in female and male, and log2 of female over male expression, for the different macrochromosomes of pygmy rattlesnake using different cutoff values. Only genes with FPKM values over 0 (upper panel), 1 (middle panel), or 10 (lower panel) were used in the analysis. (PDF) [file pbio.1001643.s014.pdf]

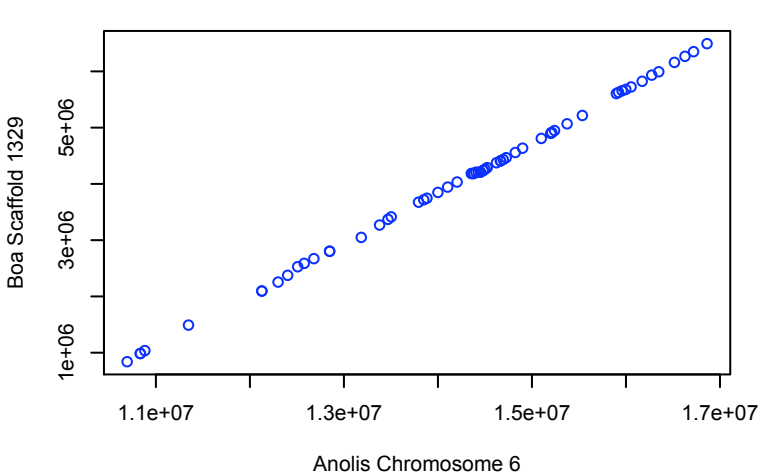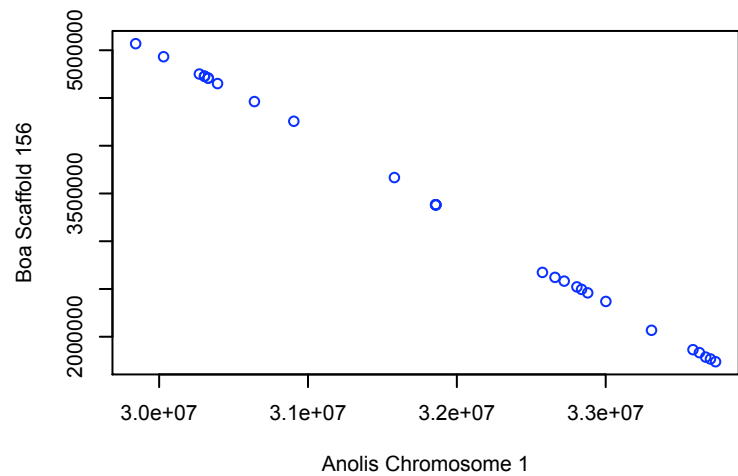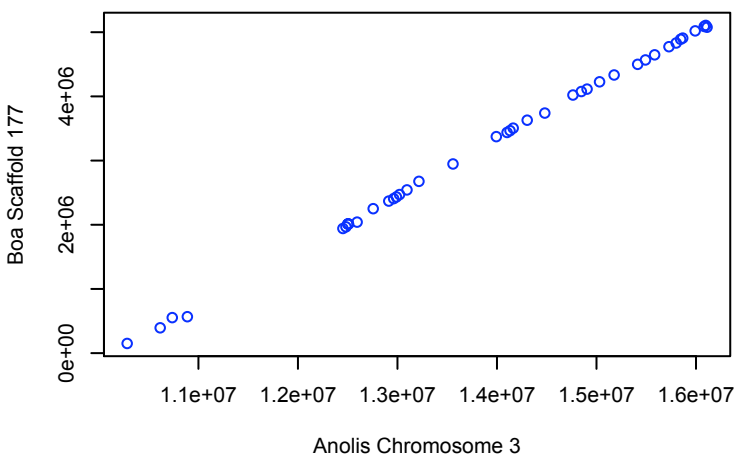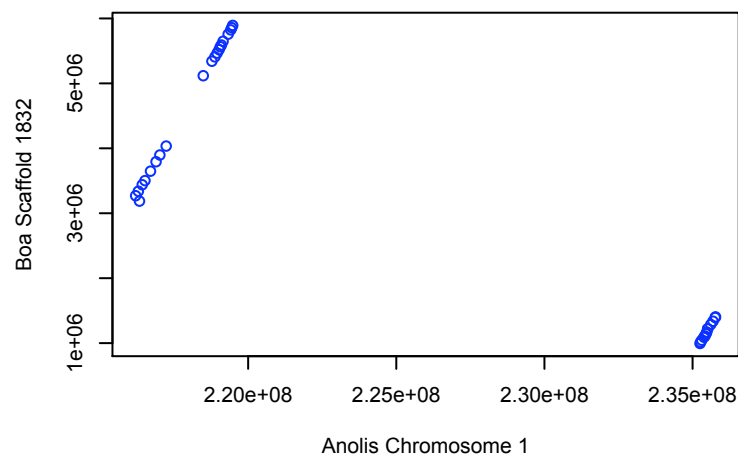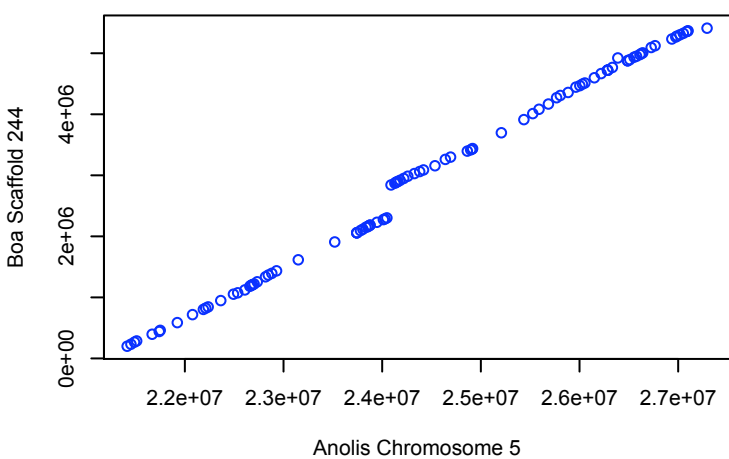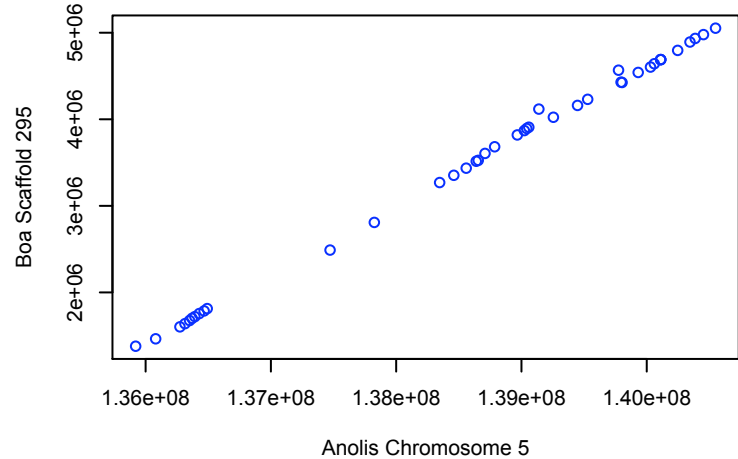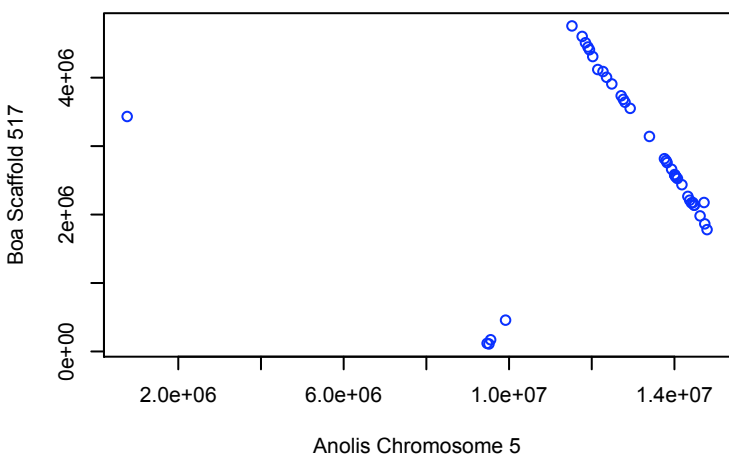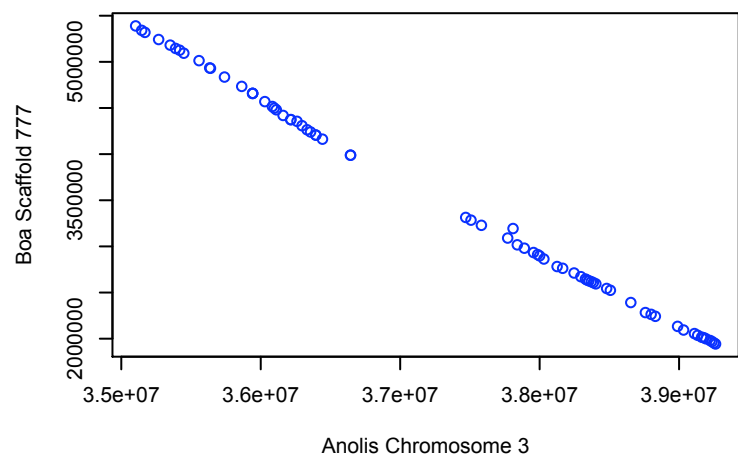

Supplement: Figure S13 — Synteny of genes between Anolis chromosomes and the eight largest boa scaffolds that mapped to macrochromosomes. Anolis CDS sequences were mapped to the boa genomic scaffolds using blat. The corresponding location of each gene on the eight largest scaffolds that mapped to Anolis macrochromosomes was plotted against their location on the corresponding Anolis chromosome. (PDF) [file pbio.1001643.s015.pdf]

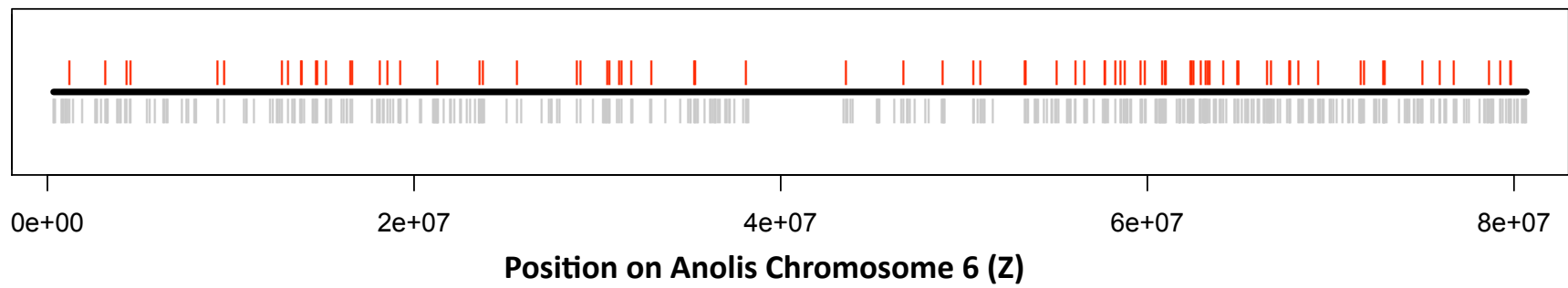

Supplement: Figure S14 — Location of all 431 Z-linked pygmy rattlesnake transcripts (grey) and of the 80 genes with log2(F/M expression) = 0±0.3 (red, corresponds approximately to 0.8<F/M<1.2) along chromosome 6 (Z). (PDF) [file pbio.1001643.s016.pdf]
