# Supplementary material for: Comparative Sex Chromosome Genomics in Snakes: Differentiation, Evolutionary Strata, and Lack of Global Dosage Compensation
Source: PLoS Biol. 2013 Aug 27;11(8):e1001643. doi: 10.1371/journal.pbio.1001643 (PMC3754893; doi:10.1371/journal.pbio.1001643)
Supplement: Table S1 — Assembly statistics for boa, pygmy rattlesnake, and garter snake genomes. (DOCX) [file pbio.1001643.s017.docx]

**Table S1**. Assembly statistics for boa, garter snake and pygmy rattlesnake genomes.

| Species | N50 (bp) | Assembled Genome Size | Number scaffolds | Comment |
| --- | --- | --- | --- | --- |
| Boa | 16,487 | 1,415,225,749 | 144,256 | Female library only |
| Garter snake | 11,744 | 1,458,958,631 | 217,987 | Libraries for both sexes |
| Pygmy rattlesnake | 12,501 | 1,309,022,726 | 187,303 | Libraries for both sexes |
