# Supplementary material for: Comparative Sex Chromosome Genomics in Snakes: Differentiation, Evolutionary Strata, and Lack of Global Dosage Compensation
Source: PLoS Biol. 2013 Aug 27;11(8):e1001643. doi: 10.1371/journal.pbio.1001643 (PMC3754893; doi:10.1371/journal.pbio.1001643)
Supplement: Table S2 — Mapping of known Z-linked and autosomal markers of rat snake to the Anolis genome. (DOCX) [file pbio.1001643.s018.docx]

**Table S2**. Mapping of known Z-linked and autosomal markers of rat snake to the *Anolis* genome.

| *Anolis* genome | rat snake Z-linked | rat snake autosomal |
| --- | --- | --- |
| Chr. 1 | 0 | 12 |
| Chr. 2 | 0 | 14 |
| Chr. 3 | 0 | 9 |
| Chr. 4 | 0 | 13 |
| Chr. 5 | 0 | 5 |
| Chr. 6 | 9 | 1 |
| Unknown | 2 | 38 |
| Other | 0 | 1 |
