# Supplementary material for: Comparative Sex Chromosome Genomics in Snakes: Differentiation, Evolutionary Strata, and Lack of Global Dosage Compensation
Source: PLoS Biol. 2013 Aug 27;11(8):e1001643. doi: 10.1371/journal.pbio.1001643 (PMC3754893; doi:10.1371/journal.pbio.1001643)
Supplement: Table S3 — Average pairwise synonymous and non-synonymous divergence between all the species used in this study. (DOCX) [file pbio.1001643.s019.docx]

**Table S3.** Pairwise species divergence (Nei-Gojobori)

| Pairwise Ks values (Chr. 1-5, Nei-Gojobori) | | | | |
| --- | --- | --- | --- | --- |
|  | Boa | Pygmy Rattlesnake | Garter snake | Anolis |
| Boa |  | 0.227 | 0.245 | 0.700 |
| Pygmy Rattlesnake | 0.263 |  | 0.172 | 0.779 |
| Garter snake | 0.286 | 0.228 |  | 0.801 |
| Anolis | 0.726 | 0.779 | 0.848 |  |
|  |  |  |  |  |
| Pairwise Ka values (Chr. 1-5, Nei-Gojobori) | | | | |
|  | Boa | Pygmy Rattlesnake | Garter snake | Anolis |
| Boa |  | 0.032 | 0.035 | 0.062 |
| Pygmy Rattlesnake | 0.054 |  | 0.028 | 0.075 |
| Garter snake | 0.059 | 0.065 |  | 0.077 |
| Anolis | 0.077 | 0.100 | 0.103 |  |
|  |  |  |  |  |
| Pairwise Ka/Ks values (Chr. 1-5, Nei-Gojobori) | | | | |
|  | Boa | Pygmy Rattlesnake | Garter snake | Anolis |
| Boa |  | 0.129 | 0.133 | 0.085 |
| Pygmy Rattlesnake | 0.164 |  | 0.157 | 0.091 |
| Garter snake | 0.166 | 0.200 |  | 0.092 |
| Anolis | 0.106 | 0.114 | 0.115 |  |
|  |  |  |  |  |
| Pairwise Ks values (Chr. 6(Z), Nei-Gojobori) | | | | |
|  | Boa | Pygmy Rattlesnake | Garter snake | Anolis |
| Boa |  | 0.259 | 0.277 | 0.733 |
| Pygmy Rattlesnake | 0.301 |  | 0.188 | 0.853 |
| Garter snake | 0.308 | 0.254 |  | 0.853 |
| Anolis | 0.763 | 0.910 | 0.908 |  |
|  |  |  |  |  |
| Pairwise Ka values (Chr. 6(Z), Nei-Gojobori) | | | | |
|  | Boa | Pygmy Rattlesnake | Garter snake | Anolis |
| Boa |  | 0.039 | 0.041 | 0.057 |
| Pygmy Rattlesnake | 0.070 |  | 0.037 | 0.085 |
| Garter snake | 0.073 | 0.090 |  | 0.082 |
| Anolis | 0.074 | 0.113 | 0.117 |  |
|  |  |  |  |  |
| Pairwise Ka/Ks values (Chr. 6(Z), Nei-Gojobori) | | | | |
|  | Boa | Pygmy Rattlesnake | Garter snake | Anolis |
| Boa |  | 0.135 | 0.140 | 0.077 |
| Pygmy Rattlesnake | 0.183 |  | 0.185 | 0.094 |
| Garter snake | 0.176 | 0.237 |  | 0.094 |
| Anolis | 0.096 | 0.115 | 0.122 |  |

grey shaded area=median; no shading=mean.
