# Supplementary material for: Comparative Sex Chromosome Genomics in Snakes: Differentiation, Evolutionary Strata, and Lack of Global Dosage Compensation
Source: PLoS Biol. 2013 Aug 27;11(8):e1001643. doi: 10.1371/journal.pbio.1001643 (PMC3754893; doi:10.1371/journal.pbio.1001643)
Supplement: Table S4 — Divergence at synonymous and nonsynonymous sites between Z-W gametologs. (DOCX) [file pbio.1001643.s020.docx]

**Table S4.** Divergence (Nei-Gojobori) between ZW gametologs

| Gene | Location on Anolis Chr.6 | Ka | Ks | Ka/Ks | Length | S-Sites | N-Sites | S-Subst. | N-Subst. |
| --- | --- | --- | --- | --- | --- | --- | --- | --- | --- |
| Pygmy rattlesnake Z-W divergence | | | | | | | | | |
| ENSACAG00000002768 | 69408108 | 0.0106958 | 0.359612 | 0.0297426 | 363 | 80.5118 | 282.488 | 23 | 3 |
| ENSACAG00000002858 | 8017131 | 0.0691229 | 0.182602 | 0.378544 | 327 | 77.1259 | 249.874 | 12.5 | 16.5 |
| ENSACAG00000003160 | 68937100 | 0.0539067 | 0.213836 | 0.252094 | 123 | 26.8739 | 96.1261 | 5 | 5 |
| ENSACAG00000004618 | 78374323 | 0.0234671 | 0.238961 | 0.0982047 | 111 | 24.434 | 86.566 | 5 | 2 |
| ENSACAG00000005126 | 77086085 | 0.218532 | NA | NA | 81 | 20.3377 | 60.6623 | 15.5 | 11.5 |
| ENSACAG00000006615 | 74968000 | 0.0134495 | 0.215978 | 0.0622726 | 198 | 47.9585 | 150.042 | 9 | 2 |
| ENSACAG00000006793 | 74737400 | NA | 0.286166 | 0 | 105 | 25.2206 | 79.7794 | 6 | NA |
| ENSACAG00000007348 | 64781200 | 0.036893 | 0.44285 | 0.0833081 | 144 | 32.8899 | 111.11 | 11 | 4 |
| ENSACAG00000009052 | 16356100 | 0.0691989 | 0.0720741 | 0.960107 | 135 | 29.1039 | 105.896 | 2 | 7 |
| ENSACAG00000009562 | 58571400 | 0.145701 | 0.20539 | 0.70939 | 126 | 27.8293 | 98.1707 | 5 | 13 |
| ENSACAG00000012203 | 64097400 | 0.0767549 | 0.299512 | 0.256267 | 276 | 56.6957 | 219.304 | 14 | 16 |
| ENSACAG00000013955 | 80565890 | 0.0379901 | 0.286201 | 0.132739 | 102 | 21.0151 | 80.9849 | 5 | 3 |
| ENSACAG00000014008 | 80604000 | 0.0445011 | 0.440984 | 0.100913 | 213 | 50.9877 | 162.012 | 17 | 7 |
| ENSACAG00000014046 | 80673200 | 0.0602274 | 0.126653 | 0.47553 | 138 | 34.324 | 103.676 | 4 | 6 |
| ENSACAG00000016321 | 64979634 | 0.0165149 | 0.29338 | 0.0562918 | 372 | 65.8971 | 306.103 | 16 | 5 |
| ENSACAG00000016584 | 65510130 | 0.0426319 | 0.168669 | 0.252754 | 123 | 26.4816 | 96.5184 | 4 | 4 |
| ENSACAG00000016868 | 66062800 | 0.0716311 | 0.325779 | 0.219877 | 201 | 47.3043 | 153.696 | 12.5 | 10.5 |
| ENSACAG00000017889 | 71207800 | 0.128623 | 1.80788 | 0.0711454 | 231 | 56.1518 | 174.848 | 38.3 | 20. 7 |
| ENSACAG00000017951 | 72660000 | 0.0330126 | 0.568005 | 0.0581203 | 477 | 105.444 | 371.556 | 42 | 12 |
| ENSACAG00000023169 | 16339728 | 0.0200796 | 0.29871 | 0.067221 | 192 | 40.5854 | 151.415 | 10 | 3 |
| ENSACAG00000023742 | 67773115 | 0.0767128 | 0.191462 | 0.400669 | 414 | 91.7287 | 322.271 | 15.5 | 23.5 |
| ENSACAG00000028153 | 80188775 | 0.0966501 | 0.159602 | 0.605568 | 123 | 34.7793 | 88.2207 | 5 | 8 |
| Garter snake Z-W divergence | | | | | | | | | |
| ENSACAG00000000628 | 10882500 | 0.00704103 | 0.322829 | 0.0218104 | 177 | 34.3076 | 142.692 | 9 | 1 |
| ENSACAG00000001166 | 9656307 | 0.0383619 | 0.320683 | 0.119626 | 237 | 49.8209 | 187.179 | 13 | 7 |
| ENSACAG00000002847 | 69325390 | 0.13391 | 0.332945 | 0.4022 | 138 | 34.7138 | 103.286 | 9.33333 | 12.6667 |
| ENSACAG00000006793 | 74737400 | 0.0604949 | 0.321931 | 0.187913 | 399 | 102.198 | 296.802 | 26.75 | 17.25 |
| ENSACAG00000007418 | 73958082 | 0.0200271 | 0.478804 | 0.0418273 | 159 | 32.4951 | 126.505 | 11.5 | 2.5 |
| ENSACAG00000007583 | 13847667 | 0.0240907 | 0.174338 | 0.138184 | 294 | 76.0699 | 217.93 | 11.8333 | 5.16667 |
| ENSACAG00000009052 | 16356100 | 0.0408203 | 0.191366 | 0.21331 | 171 | 32.5631 | 138.437 | 5.5 | 5.5 |
| ENSACAG00000009416 | 338359 | NA | 0.362099 | 0 | 129 | 27.8543 | 101.146 | 8 | NA |
| ENSACAG00000013405 | 80214900 | NA | 0.277866 | 0 | 360 | 86.1318 | 273.868 | 20 | NA |
| ENSACAG00000016868 | 66062800 | 0.0192341 | 0.322901 | 0.0595666 | 132 | 26.679 | 105.321 | 7 | 2 |
| ENSACAG00000017541 | 67791874 | 0.0568255 | 0.472386 | 0.120295 | 165 | 37.0903 | 127.91 | 13 | 7 |
| ENSACAG00000028153 | 80188775 | 0.244657 | 0.436597 | 0.560373 | 144 | 32.2286 | 111.771 | 10.6667 | 23.3333 |
