# Supplementary material for: Comparative Sex Chromosome Genomics in Snakes: Differentiation, Evolutionary Strata, and Lack of Global Dosage Compensation
Source: PLoS Biol. 2013 Aug 27;11(8):e1001643. doi: 10.1371/journal.pbio.1001643 (PMC3754893; doi:10.1371/journal.pbio.1001643)
Supplement: Table S5 — Synonymous divergence Ks between ZW paralogs using three other models to estimate Ks. (DOCX) [file pbio.1001643.s021.docx]

**Table S5** Ks between ZW paralogs using three other models to estimate Ks, as implemented in KaKs_calculator.

| Pygmy Rattlesnake | LPB | | | LWL | | | YN | | |
| --- | --- | --- | --- | --- | --- | --- | --- | --- | --- |
|  | Ka | Ks | Ka/Ks | Ka | Ks | Ka/Ks | Ka | Ks | Ka/Ks |
| ENSACAG00000002768 | 0.015 | 0.218 | 0.070 | 0.014 | 0.364 | 0.039 | 0.012 | 0.274 | 0.043 |
| ENSACAG00000002858 | 0.072 | 0.154 | 0.464 | 0.067 | 0.202 | 0.330 | 0.072 | 0.167 | 0.430 |
| ENSACAG00000003160 | 0.059 | 0.175 | 0.339 | 0.053 | 0.229 | 0.232 | 0.064 | 0.135 | 0.476 |
| ENSACAG00000004618 | 0.025 | 0.150 | 0.164 | 0.023 | 0.259 | 0.089 | 0.024 | 0.205 | 0.119 |
| ENSACAG00000005126 | 0.216 | 0.441 | 0.489 | 0.225 | 0.719 | 0.312 | 0.214 | 2.264 | 0.094 |
| ENSACAG00000006615 | 0.023 | 0.142 | 0.159 | 0.020 | 0.202 | 0.099 | 0.015 | 0.158 | 0.096 |
| ENSACAG00000006793 | NA | 0.256 | 0.000 | NA | 0.286 | 0.000 | NA | 0.339 | 0.000 |
| ENSACAG00000007348 | 0.036 | 0.372 | 0.097 | 0.036 | 0.453 | 0.081 | 0.037 | 0.450 | 0.083 |
| ENSACAG00000009052 | 0.080 | 0.043 | 1.886 | 0.068 | 0.081 | 0.845 | 0.072 | 0.066 | 1.083 |
| ENSACAG00000009562 | 0.165 | 0.156 | 1.060 | 0.143 | 0.253 | 0.565 | 0.148 | 0.224 | 0.659 |
| ENSACAG00000012203 | 0.092 | 0.225 | 0.408 | 0.080 | 0.302 | 0.263 | 0.098 | 0.161 | 0.612 |
| ENSACAG00000013955 | 0.055 | 0.144 | 0.384 | 0.052 | 0.250 | 0.208 | 0.040 | 0.244 | 0.165 |
| ENSACAG00000014008 | 0.048 | 0.373 | 0.128 | 0.044 | 0.441 | 0.100 | 0.051 | 0.306 | 0.166 |
| ENSACAG00000014046 | 0.068 | 0.112 | 0.603 | 0.060 | 0.135 | 0.445 | 0.069 | 0.095 | 0.723 |
| ENSACAG00000016321 | 0.019 | 0.146 | 0.127 | 0.016 | 0.310 | 0.053 | 0.018 | 0.189 | 0.097 |
| ENSACAG00000016584 | 0.047 | 0.180 | 0.259 | 0.042 | 0.194 | 0.217 | 0.048 | 0.121 | 0.394 |
| ENSACAG00000016868 | 0.075 | 0.265 | 0.282 | 0.069 | 0.353 | 0.196 | 0.078 | 0.251 | 0.312 |
| ENSACAG00000017889 | 0.135 | 1.573 | 0.086 | 0.133 | 1.572 | 0.084 | 0.090 | 3.498 | 0.026 |
| ENSACAG00000017951 | 0.036 | 0.389 | 0.093 | 0.034 | 0.573 | 0.059 | 0.036 | 0.444 | 0.082 |
| ENSACAG00000023169 | 0.028 | 0.196 | 0.143 | 0.026 | 0.294 | 0.089 | 0.022 | 0.230 | 0.095 |
| ENSACAG00000023742 | 0.082 | 0.167 | 0.494 | 0.073 | 0.213 | 0.343 | 0.084 | 0.155 | 0.540 |
| ENSACAG00000028153 | 0.104 | 0.164 | 0.635 | 0.091 | 0.181 | 0.503 | 0.114 | 0.118 | 0.968 |
|  |  |  |  |  |  |  |  |  |  |
| Garter Snake | LPB | | | LWL | | | YN | | |
|  | Ka | Ks | Ka/Ks | Ka | Ks | Ka/Ks | Ka | Ks | Ka/Ks |
| ENSACAG00000000628 | 0.015 | 0.210 | 0.071 | 0.016 | 0.278 | 0.058 | NA | 0.294 | 0.000 |
| ENSACAG00000001166 | 0.045 | 0.274 | 0.163 | 0.038 | 0.341 | 0.112 | 0.044 | 0.213 | 0.206 |
| ENSACAG00000001904 | 0.050 | 1.839 | 0.027 | 0.045 | 1.837 | 0.025 | 0.037 | 2.330 | 0.016 |
| ENSACAG00000002847 | 0.149 | 0.313 | 0.477 | 0.134 | 0.348 | 0.385 | 0.166 | 0.209 | 0.797 |
| ENSACAG00000006793 | 0.063 | 0.276 | 0.228 | 0.060 | 0.341 | 0.175 | 0.065 | 0.269 | 0.242 |
| ENSACAG00000007418 | 0.020 | 0.393 | 0.051 | 0.018 | 0.515 | 0.035 | 0.018 | 0.386 | 0.046 |
| ENSACAG00000007583 | 0.027 | 0.143 | 0.187 | 0.023 | 0.184 | 0.127 | 0.024 | 0.179 | 0.133 |
| ENSACAG00000009052 | 0.049 | 0.113 | 0.431 | 0.048 | 0.170 | 0.279 | 0.040 | 0.205 | 0.193 |
| ENSACAG00000009416 | NA | 0.300 | 0.000 | NA | 0.369 | 0.000 | NA | 0.260 | 0.000 |
| ENSACAG00000013405 | NA | 0.226 | 0.000 | NA | 0.292 | 0.000 | NA | 0.224 | 0.000 |
| ENSACAG00000016868 | 0.018 | 0.246 | 0.072 | 0.014 | 0.359 | 0.040 | 0.020 | 0.304 | 0.065 |
| ENSACAG00000017541 | 0.067 | 0.337 | 0.198 | 0.057 | 0.480 | 0.119 | 0.067 | 0.305 | 0.220 |
| ENSACAG00000028153 | 0.260 | 0.370 | 0.701 | 0.249 | 0.429 | 0.579 | 0.275 | 0.319 | 0.862 |

LPB Li, W.H. (1993) and Pamilo, P. and Bianchi, N.O. (1993)

LWL Li, W.H., et al. (1985)

YN Yang, Z. and Nielsen, R. (2000)
