# Supplementary material for: Comparative Sex Chromosome Genomics in Snakes: Differentiation, Evolutionary Strata, and Lack of Global Dosage Compensation
Source: PLoS Biol. 2013 Aug 27;11(8):e1001643. doi: 10.1371/journal.pbio.1001643 (PMC3754893; doi:10.1371/journal.pbio.1001643)
Supplement: Table S6 — List of confirmed and putative W-linked transcripts in pygmy rattlesnake. Female and male RNA-seq and DNA-seq reads were mapped to the de novo transcriptome using Bowtie2, and the resulting alignments were used to estimate expression levels (FPKM, with Cufflinks) and genomic male and female read counts. Transcripts with male/female read counts below 0.1 and female-specific expression were classified as putative W-derived sequences (only transcripts with at least 30 genomic female reads were considered in this analysis). The PCR confirmation of W-linkage of some of the transcripts is described in Figure S11. (DOCX) [file pbio.1001643.s022.docx]

**Table S6:** Summary of putative pygmy rattlesnake transcripts (male/female genomic coverage<0.1, female-specific expression)

| Pygmy rattlesnake W-linked transcripts confirmed experimentally | | | |
| --- | --- | --- | --- |
| **Scaffold** | **Female FPKM** | **tblastx E-value** | **tblastx best hit** |
| C206150 | 7.03838 | 5.00E-12 | Agkistrodon contortrix clone E33DIFJ02GN6M2 microsatellite sequence |
| scaffold2801 | 16.1686 | 3.00E-11 | Xenopus (Silurana) tropicalis ubiquitin-conjugating enzyme E2M (ube2m), mRNA |
| scaffold2997 | 14.1922 | NA | No significant similarity found. |
| C235599 | 7.57759 | 0.001 | Micrurus fulvius clone FQ6DGU405FYLBQ microsatellite sequence |
| scaffold3714 | 5.86897 | 1.00E-53 | Trimeresurus flavoviridis HLP gene for HSF-like protein, partial cds, exon 1, intron 1, exon 2, L1-like LINE gene for L1-encoded reverse transcriptase-like protein, complete cds |
| scaffold7127 | 9.87032 | 1.00E-71 | Fejervarya limnocharis isolate NA0083 28S ribosomal RNA gene, partial sequence |
|  |  |  |  |
| Putative pygmy rattlesnake W-linked transcripts, not tested experimentally | | | |
| **Scaffold** | **Female FPKM** | **tblastx E-value** | **tblastx best hit** |
| scaffold5225 | 3.85042 | NA | No significant similarity found*** |
|  | | | |
| Parasite-derived transcript | | | |
| **Scaffold** | **Female FPKM** | **tblastx E-value** | **tblastx best hit** |
| C216554 | 92.4672 | 2.00E-16 | Goussia balatonica strain 608 28S ribosomal RNA gene, partial sequence |
| scaffold1679 | 6.12184 | 4.00E-81 | Hepatozoon sp. BV1 18S ribosomal RNA gene, partial sequence |
| scaffold4937 | 47.4848 | 3.00E-139 | Sarcocystis rileyi 28S ribosomal RNA gene, partial sequence |
|  |  |  |  |
| Putative W-linked transcripts shown to be autosomal | | | |
| **Scaffold** | **Female FPKM** | **tblastx E-value** | **tblastx best hit** |
| C215941 | 6.13881 | 0.046 | Trimeresurus flavoviridis vascular endothelial growth factor A190 isoform precursor, gene, complete cds |
| scaffold488 | 27.2288 | 1.00E-20 | Pantherophis guttatus HOXC13 (HoxC13) gene, complete cds; and HOXC12 (HoxC12) gene, partial cds |

***mainly composed of CTTT repeats
