# Supplementary material for: Comparative Sex Chromosome Genomics in Snakes: Differentiation, Evolutionary Strata, and Lack of Global Dosage Compensation
Source: PLoS Biol. 2013 Aug 27;11(8):e1001643. doi: 10.1371/journal.pbio.1001643 (PMC3754893; doi:10.1371/journal.pbio.1001643)
Supplement: Table S7 — Detecting male-driven and faster-Z evolution using three other models to estimate Ka and Ks. Median Ka, Ks, and Ka/Ks values for boa-Anolis, pygmy rattlesnake-Anolis and garter snake-Anolis comparisons are given for autosomal macrochromosomes and for chromosome 6/Z. All estimates of rates of evolution were obtained using KaKs_calculator. (DOCX) [file pbio.1001643.s023.docx]

**Table S7** Detecting male-driven and faster-Z evolution using three other models to estimate Ka and Ks.

| **Ka** | Boa-Anolis | | Pygmy-Anolis | | Garter-Anolis | |  |  |  |
| --- | --- | --- | --- | --- | --- | --- | --- | --- | --- |
| Model | Autosomes | Z | Autosomes | Z | Autosomes | Z |  |  |  |
| LPB | 0.067 | 0.063 | 0.080 | 0.089 | 0.082 | 0.092 |  |  |  |
| LWL | 0.063 | 0.059 | 0.076 | 0.085 | 0.078 | 0.085 |  |  |  |
| YN | 0.063 | 0.059 | 0.075 | 0.085 | 0.076 | 0.086 |  |  |  |
| **Ka** | Pygmy-Anolis / Boa-Anolis | | P-value | Garter-Anolis / Boa-Anolis | | P-value | Pygmy-Anolis / Garter-Anolis | | P-value |
| Model | Autosomes | Z | (Auto vs Z) | Autosomes | Z | (Auto vs Z) | Autosomes | Z | (Auto vs Z) |
| LPB | 1.185 | 1.423 | **0.000** | 1.215 | 1.460 | **0.000** | 1.025 | 1.026 | 0.361 |
| LWL | 1.194 | 1.424 | **0.000** | 1.226 | 1.432 | **0.000** | 1.028 | 1.006 | 0.321 |
| YN | 1.186 | 1.453 | **0.000** | 1.206 | 1.458 | **0.000** | 1.017 | 1.003 | 0.431 |
|  |  |  |  |  |  |  |  |  |  |
| **Ks** | Boa-Anolis |  | Pygmy-Anolis |  | Garter-Anolis |  |  |  |  |
| Model | Autosomes | Z | Autosomes | Z | Autosomes | Z |  |  |  |
| LPB | 0.547 | 0.575 | 0.607 | 0.644 | 0.619 | 0.667 |  |  |  |
| LWL | 0.682 | 0.707 | 0.753 | 0.809 | 0.771 | 0.825 |  |  |  |
| YN | 0.636 | 0.664 | 0.745 | 0.798 | 0.775 | 0.865 |  |  |  |
| **Ks** | Pygmy-Anolis / Boa-Anolis | | P-value | Garter-Anolis / Boa-Anolis | | P-value | Pygmy-Anolis / Garter-Anolis | | P-value |
| Model | Autosomes | Z | (Auto vs Z) | Autosomes | Z | (Auto vs Z) | Autosomes | Z | (Auto vs Z) |
| LPB | 1.109 | 1.119 | **0.000** | 1.131 | 1.160 | **0.000** | 1.020 | 1.036 | 0.510 |
| LWL | 1.104 | 1.144 | **0.000** | 1.129 | 1.167 | **0.000** | 1.023 | 1.020 | 0.854 |
| YN | 1.172 | 1.202 | **0.000** | 1.220 | 1.303 | **0.002** | 1.041 | 1.084 | 0.433 |
|  |  |  |  |  |  |  |  |  |  |
| **Ka/Ks** | Boa-Anolis | | Pygmy-Anolis | | Garter-Anolis | |  |  |  |
| Model | Autosomes | Z | Autosomes | Z | Autosomes | Z |  |  |  |
| LPB | 0.121 | 0.114 | 0.132 | 0.137 | 0.132 | 0.139 |  |  |  |
| LWL | 0.092 | 0.086 | 0.099 | 0.102 | 0.100 | 0.106 |  |  |  |
| YN | 0.093 | 0.080 | 0.092 | 0.095 | 0.092 | 0.099 |  |  |  |
| **Ka/Ks** | Pygmy-Anolis / Boa-Anolis | | P-value | Garter-Anolis / Boa-Anolis | | P-value | Pygmy-Anolis / Garter-Anolis | | P-value |
| Model | Autosomes | Z | (Auto vs Z) | Autosomes | Z | **(Auto vs Z)** | Autosomes | Z | (Auto vs Z) |
| LPB | 1.083 | 1.201 | **0.000** | 1.085 | 1.219 | **0.000** | 1.001 | 1.015 | 0.245 |
| LWL | 1.080 | 1.187 | **0.000** | 1.085 | 1.239 | **0.000** | 1.005 | 1.044 | 0.353 |
| YN | 0.987 | 1.185 | **0.000** | 0.985 | 1.234 | **0.000** | 0.999 | 1.042 | 0.663 |

LPB Li, W.H. (1993) and Pamilo, P. and Bianchi, N.O. (1993)

LWL Li, W.H., et al. (1985)

YN Yang, Z. and Nielsen, R. (2000)
