# Supplementary material for: Comparative Sex Chromosome Genomics in Snakes: Differentiation, Evolutionary Strata, and Lack of Global Dosage Compensation
Source: PLoS Biol. 2013 Aug 27;11(8):e1001643. doi: 10.1371/journal.pbio.1001643 (PMC3754893; doi:10.1371/journal.pbio.1001643)
Supplement: Table S8 — Number of RNA-seq and DNA-seq reads generated for/used in this analysis. (DOCX) [file pbio.1001643.s024.docx]

**Table S8** Number of RNA-seq and DNA-seq reads generated for/used in this analysis

| **Sample** | **RNA-seq** | **DNA-seq** |
| --- | --- | --- |
| Pygmy Rattlesnake Female | 39,887,546 | 105,176,638 |
| Pygmy Rattlesnake Male | 38,950,754 | 93,965,472 |
| Boa Female | 25,971,656 | 137,326,690 |
| Boa Male | 25,971,656 | 141,081,150* |
| Garter Snake Female | No RNA-seq data | 48,159,317 |
| Garter Snake Male | No RNA-seq data | 56,733,501 |

*Downloaded from the Assemblathon project
